# Genetic affinities between the Yami tribe people of Orchid Island and the Philippine Islanders of the Batanes archipelago

**DOI:** 10.1186/1471-2156-12-21

**Published:** 2011-01-31

**Authors:** Jun-Hun Loo, Jean A Trejaut, Ju-Chen Yen, Zong-Sian Chen, Chien-Liang Lee, Marie Lin

**Affiliations:** 1 Transfusion Medicine and Molecular Anthropology Research Laboratory, Department of Medical Research, Mackay Memorial Hospital, Taipei, Taiwan; 2 Department and Graduate Institute of Forensic Medicine College of Medicine, National Taiwan University, Taipei, Taiwan

## Abstract

**Background:**

Yami and Ivatan islanders are Austronesian speakers from Orchid Island and the Batanes archipelago that are located between Taiwan and the Philippines. The paternal genealogies of the Yami tribe from 1962 monograph of Wei and Liu were compared with our dataset of non-recombining Y (NRY) chromosomes from the corresponding families. Then mitochondrial DNA polymorphism was also analyzed to determine the matrilineal relationships between Yami, Ivatan, and other East Asian populations.

**Results:**

The family relationships inferred from the NRY Phylogeny suggested a low number of paternal founders and agreed with the genealogy of Wei and Liu (P < 0.01). Except for one Y short tandem repeat lineage (Y-STR), seen in two unrelated Yami families, no other Y-STR lineages were shared between villages, whereas mtDNA haplotypes were indiscriminately distributed throughout Orchid Island.

The genetic affinity seen between Yami and Taiwanese aborigines or between Ivatan and the Philippine people was closer than that between Yami and Ivatan, suggesting that the Orchid islanders were colonized separately by their nearest neighbors and bred in isolation. However a northward gene flow to Orchid Island from the Philippines was suspected as Yami and Ivatan peoples both speak Western Malayo-Polynesian languages which are not spoken in Taiwan. Actually, only very little gene flow was observed between Yami and Ivatan or between Yami and the Philippines as indicated by the sharing of mtDNA haplogroup B4a1a4 and one O1a1* Y-STR lineage.

**Conclusions:**

The NRY and mtDNA genetic information among Yami tribe peoples fitted well the patrilocal society model proposed by Wei and Liu. In this proposal, there were likely few genetic exchanges among Yami and the Philippine people. Trading activities may have contributed to the diffusion of Malayo-Polynesian languages among them.

Finally, artifacts dating 4,000 YBP, found on Orchid Island and indicating association with the Out of Taiwan hypothesis might be related to a pioneering stage of settlement, as most dating estimates inferred from DNA variation in our data set ranged between 100-3,000 YBP.

## Background

**Figure 1:**
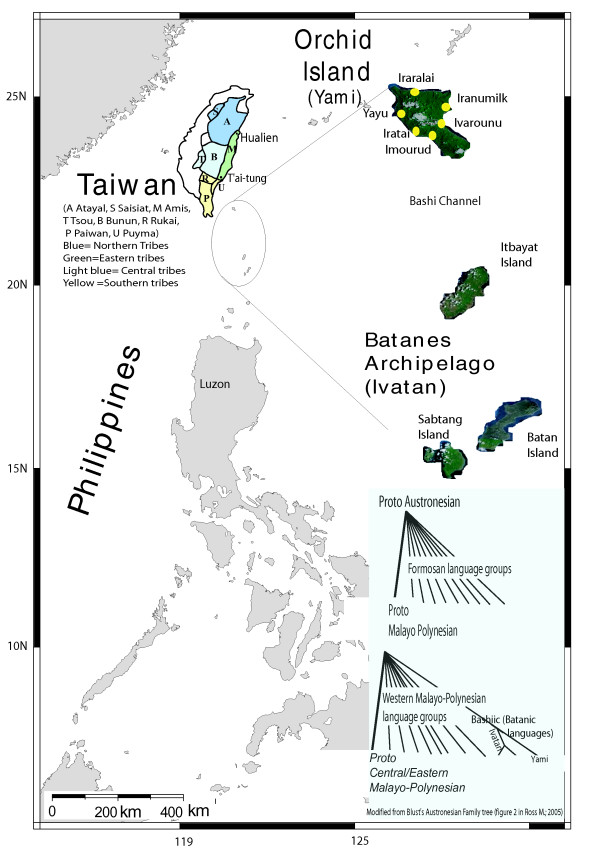
**Location of Orchid Island and the Batanes archipelago**. Insert shows the upper nodes of the Austronesian family tree based on the work of Blust (1977, [3])

Orchid Island, is located 49 nautical miles from the southeast coast of Taiwan along the Bashi (or Luzon) channel in the Pacific Ocean, and is home to the Yami tribe (also known as Tao). The Ivatan tribe people are inhabitants of Itbayat in the Batanes archipelago which is south of Orchid Island (Figure 1). The languages of Yami and Ivatan belongs to the Batanic sub-branch of western Malayo-Polynesian languages (Figure 1), which also belongs to the 10^th ^branch of the Austronesian (AN) languages group [1,2]. The Yami are the only non-Formosan Austronesian speakers among Taiwan Aborigines (TwA) [3]. They also have a close cultural relationship with the Ivatan. According to an oral folk tale, the Yamis believe that their ancestors came from the Batanes archipelago [4].

The archaeological findings in Orchid Island have shown evidence of Fine Corded Ware Culture, which is related to the Peinan culture [5]. These middle Neolithic artifacts were found on the east coast of Taiwan between Hualien and Taitung (Figure 1). These findings indicate contact and possibly migration from Taiwan to Orchid Island ~4,000 years before present (YBP). Furthermore, the post-Neolithic oral history (~1,500 YBP), reports that the interactions between Orchid island and the Batanes archipelago islanders were frequent until ~300 YBP [6], but the interactions between TwA and Orchid Islanders have ceased much earlier.

The archaeological excavation from Batanes in 2002 [7] showed that the Batanes archipelago had been inhabited 4000 YBP. Similar to Orchid Island findings [8], the sites in Batanes indicated connections with middle to late Neolithic cultures originated in the eastern coast of Taiwan. More recently, two very specific forms of ear pendants that were made of green nephrite from eastern Taiwan were discovered in Orchid Island and Batanes along with other artifacts dating back to 2,500 to 1500 YBP [8]. Similar artifacts of same period have been reported in Orchid Island, Batanes, the Philippines, East Malaysia, southern Vietnam, and Thailand [8]. All these findings clearly suggest prehistoric trading activities around the China seas. Carbon dating from food debris suggests that the colonization of Batanes might have happened much later (~2,500 YBP), however the dating obtained from pottery residues or inferred from Northern Luzon findings suggests 4,000 YBP [9]. These date estimates have raised questions about the relationship between the present inhabitants of Orchid Island and Batanes and the simple "stepping stone" (or "Out of Taiwan") hypothesis [10].

From the end of the 19^th ^century to the middle of the 20^th ^century, Japanese anthropologists have conducted important ethnological studies on all Taiwan Aborigines tribes, including the Yami of Orchid Island [11-20]. Inter-village cultural variations among the Yamis were first noticed by Kano [21]. However, more recent anthropological studies suggest that sharing of common attributes among villages had been overestimated and accordingly, much more variation among villages should be expected [4]. A 1962 monograph about the social structure of the Yami [22] described the paternal genealogies of a number of the Yami families, some of which would be traced back to ten generations. Wei and Liu showed that generations of same family remained in the same village. For the first stage of our study, we used Y chromosome polymorphism to determine the patrilineal relationships between Yami families and then compared the genetic analysis with the genealogical information from Wei and Liu.

In the early 18^th ^century, following a destructive typhoon and ensuing famine, 35% of the Ivatan population perished [23]. The Catholic Church arranged one fourth of the Ivatans to move south to a more sheltered island near Luzon in the Philippines. At the end of the 19^th ^century, however, many of these peoples moved back to Batanes. As a consequence, one would expect the genetic profile of the extant population of Batanes to show some similarity with the northern Luzon people in the Philippines. In 2001, a human leukocyte antigen (HLA) study showed that the HLA-A and DRB1 allele distributions of the Ivatan were similar to the Yami and to the Puyuma tribe from the southeast coast of Taiwan [24]. For the second part of this study, we use mitochondrial DNA of relevant coding regions and the control region HVS-1, together with complete mtDNA genome sequencing of the most representative haplogroups among Yami and Ivatan, to further analyze the matrilineal relationship between Yami, Ivatan, Taiwan Aborigines, the Philippine people, and other populations from Mainland and Island Southeast Asia (MSEA and ISEA).

In summary, the study proposes to test the issue of genetic stratification described by Wei and Liu using Y chromosome polymorphism. Further, using mitochondrial DNA diversity among Yami and Ivatan, we propose to examine the issue of the initial settlement of the Orchid Islands to determine whether it happened with the mid Neolithic Austronesian expansion, and whether there was gene flow between the Batanes and the Orchid Islands. We will also test the issue of genetic affinities between the Batanes and the Philippines as expected given the relocation of Batanes individuals during the XVIII century. Finally we propose to further analyze the matrilineal relationship between Yami, Ivatan, Taiwan Aborigines, the Philippine people, and other populations from mainland and island Southeast Asia (MSEA and ISEA).

## Results

### Mitochondrial DNA

The complete mtDNA sequence data of HVS-1 (nps 16,037 to 16,365), nps 8,000 to 9,000, nps 9,800 to 10,900 and nps 14,000 to 15,000 of 129 Yami and Ivatan individuals together with their detailed haplogroup classification are reported in the Additional file 1. The Yamis as determined by ten different mtDNA haplotypes showed considerably less polymorphisms than the Ivatans (20 haplotypes) or other Taiwan Aboriginal tribes (13 to 22 haplotypes) [25].

Except for haplogroup E2b1 (6.3%), all the Yami haplogroups had frequencies greater than 10%. In comparison, only four out of 15 Ivatan haplogroups exceeded 10% (total of 62%). Further, 5 haplogroups (B4a1a, B4a2a, B4c1b2, E2b1 and M7c3c) were shared between Yami and Ivatan (Table 1).

**Table 1:** mtDNA haplogroup frequencies of Ivatan, Yami and corresponding frequencies in neighboring populations

Haplogroups	China	Taiwan										Philippines	
			
	**Fujian (Han)**^**2**^ (n = 149)	**Taiwanese (Han)**^**1**^ (n = 247)	Taiwan Aborigines									**Philippines**^**$**^ (n = 323)	Ivatan (n = 50)
					
			**Atayal**^**1**^ (n = 108)	**Saisiat**^**1**^ (n = 64)	**Bunun**^**1**^ (n = 89)	**Tsou**^**1**^ (n = 60)	**Rukai**^**1**^ (n = 51)	**Paiwan**^**1**^ (n = 55)	**Puyuma**^**1**^ (n = 52)	**Amis**^**1**^ (n = 98)	Yami (n = 79)		
B4a1a		0.40	3.70			10.00	5.88			44.90		11.76	10.00
B4a1a4											15.19	0.93	4.00
B4a2a		0.81	0.93	1.56			9.80	16.36	3.85		24.05		2.00
B4c1b2	4.70	2.43				8.33		7.27	17.31		10.13	4.95	14.00
B5b1		2.02										8.67	2.00
E1a1a		0.81	0.93	28.13	16.85	8.33		3.64	5.77			10.84	12.00
E2a												3.41	2.00
E2b1			3.70	3.13	5.62					4.08	6.33		2.00
E2b2												0.31	14.00
F1a1d	0.67	0.40				10.00	5.88	1.82			22.78	0.31	
F1a3					2.25				9.61			3.72	6.00
F1a4	3.35	3.63				5.00				3.06		4.95	18.00
F4b	1.34	0.40	30.56	14.06	28.09	5.00		1.82		3.06		0.62	2.00
M7b3a		0.81	36.11	14.07	3.37		7.84			1.02	10.13	2.79	
M7b4	1.34	2.83											2.00
M7c3a						1.67				8.16			2.00
M7c3c	0.67	1.21					5.88	7.27	28.85	2.04	11.39	11.15	2.00
N9a10		0.40	1.85						1.92	7.14			6.00

Halogroup diversity	0.97	0.97	0.77	0.84	0.77	0.89	0.81	0.82	0.78	0.76	0.83	0.93	0.88

± SD	0.00	0.00	0.02	0.02	0.02	0.01	0.02	0.02	0.02	0.03	0.01	0.00	0.01

Total number of halogroups in the populations (S2)	67	76	17	13	10	11	10	11	8	12	7	43	16

Number of shared halogroups with Yami & Ivatan	3 & 5	5 & 10	3 & 6	3 & 4	2 & 4	2 & 6	4 & 3	4 & 5	3 & 6	3 & 7	7 & 5	5 & 11	5 & 16

Total Frequency shown	12.07	16.15	77.78	60.95	56.18	48.33	35.28	38.18	67.31	73.46	100	64.41	100

Tajima's D	-1.92	-1.89	-0.44	0.00	0.06	-0.15	-0.02	-0.63	0.10	-0.60	1.42	-1.50	-0.23

P value	<0.05	<0.05	>0.10	>0.10	>0.10	>0.10	>0.10	>0.10	>0.10	>0.10	>0.10	>0.10	>0.10

Sample numbers, haplogroup diversity, standard deviation [69] and number of haplogroups are obtained from the authors data set (material not shown).

Taiwanese Han includes Minnan and Hakka, these groups are not differentiable using the exact test of population differentiation using Arlequin package [55].

^@ ^No Ivatan are included in this group

^1 ^Trejaut et al. 2005; ^2 ^Trejaut unpublished data; ^3 ^Tabbada et al. 2010.

Tajima's D was obtained using DnaSp software version 5.10.01[64]; http://www.ub.es/dnasp/

Although all Yami mtDNA haplogroups were seen among TwA, some were found partially in the Philippines (Table 1). Therefore, the *F*_st _tree (additional file 2; mtDNA) posits the Yamis to be intermediate between the Ivatans and all the other Taiwanese groups (including the Amis).

**Figure 2:**
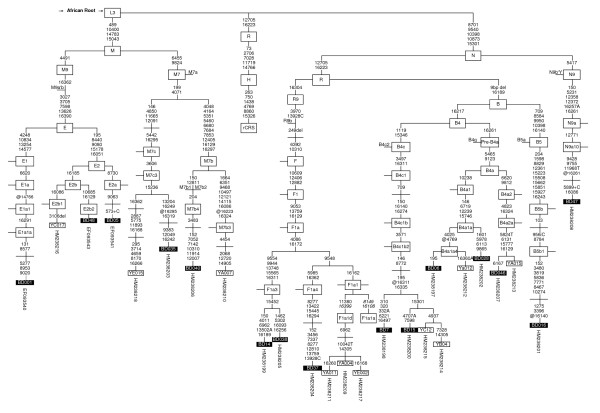
**Most parsimonious tree constructed from Yami and Ivatan complete mtDNA genome**. ┼ Open crossing along branches indicates branching reported in Van Oven 2009 @ Reverse mutation, nps 310, 315 and 16519 insertions are not indicated, Black and empty box indicate Ivatan and Yami respectively.

Complete mtDNA sequences from all phylogenetically relevant haplogroups of Yamis and Ivatans are shown in Figure 2, which include three haplogroups locally named in accordance with the van Oven "Phylotree" as F1a1d, M7b4 and N9a10 [26]. Haplogroup F1a1d [27] differs from F1a1a which was previously described by Hill et al. at np 16108 [28,29]. Nps 16399 and 11380 (Figure 2), are found in Tsou and Rukai in Taiwan (10.00% and 5.88% respectively), in Vietnam (~6%) [30,31], Fujian (<1%), and among the Yamis where drift is likely to explain the high frequency (22%) because haplotype diversity is low on the island (Table 1 and Additional file 1). The presence of these haplotypes in Yamis and near absence in the Philippines, suggests that the gene flow from Southeast Asia ended in Taiwan [30-32] and could have reached Orchid island as a result of the jade trade [8].

All B4a1a haplotypes in Yamis (15%) belonged to a sub-clade defined by nps 4025 and 16360A (Figure 2) [25]. The clade, here named B4a1a4, was not seen in Taiwan. One twig of B4a1a4 did not show np 16360A and was seen in two Ivatan individuals (4%). Further screening for the presence of np 4025 in 132 B4a1a samples was undertaken to determine the presence of B4a1a4 in other regions of Taiwan, Southeast Asia and ISEA, and if possible, to infer its origin. Five B4a1a4 lineages lacking 16360A transversion were seen among the Filipinos (1%) and three of them were different at HVS-1. The higher B4a1a4 diversity south of Orchid Island favored a Philippine origin. As indicated by the low mtDNA diversity among Yami, genetic drift must have been active on the island and most likely accounts for the high frequency of the unique B4a1a4 lineage (24%) (Table 1).

The complete mtDNA sequences and HVS-1 sequences in ISEA and Taiwan ([27] and our unpublished data) were used to estimate and compare the ages of the haplogroups found in Yami and Ivatan (Table 2). While such dates may have considerable uncertainty [33], two patterns were seen:

**Table 2:** Molecular age estimates of mtDNA haplogroups in Yami and Ivatan

	**Soares Method**				**Kivisild Method**	**Soares Method**					**Saillard Method**
		
**Haplogroup**	**ρ method obtained from all coding region synonymous mutations (transitions and transversions) of all available complete sequences**^**#**^					**ρ method obtained from all HVSI transitions between nps 16090 and 16365**^***$***^					
			
	**n**	**ρ**	**σ**	**Age of molecular variation (95% confidence interval)**	**Age of molecular variation (± variance)**		**n**	**ρ**	**σ**	**Age of molecular variation (95% confidence interval)**	**Age of molecular variation (± variance)**
		
**B4a1a**	**27**	**1.63**	**0.55**	**12,831 (4,345; 21,317)**	**11,025 ± 3,720**	**Total**	**152**	**0.59**	**0.17**	**11,132 (4,764; 17,500)**	**11,906 **± 3,431
						Yami	12	0.08	0.08	1,447 (0; 4,284)	1,614 ± 1,614
						Ivatan	7	0.08	0.08	1,551 (0; 4,591)	1,614 ± 1,614
		
**B4a1a4**	**2**	**1**	**1**	**7872 (NA)**	**NA**	**Total**	**18**	**0.14**	**0.14**	**2691 (NA)**	**2,825 **± 2,825
						Yami	12	NA^&^	NA^&^	NA^&^	NA^&^
						Ivatan	2	NA	NA	NA	NA
		
**B4a2a**	**4**	**1.5**	**0.71**	**11,808 (853; 22,763)**	**10,146 ± 4,802**	**Total**	**64**	**0.76**	**0.48**	**14,362 (3,357; 32,080)**	**15,337 **± 9,686
						Yami	19	0.04	0.04	756 (0; 2,237)	807 ± 807
						Ivatan	1	NA	NA	NA	NA
		
**B4c1b2**	**8**	**2.63**	**1.04**	**20,703 (4,657; 36,750)**	**17,789 ± 7,034**	**Total**	**98**	**0.74**	**0.21**	**13,896 (5,970; 21,823)**	**14,933 **± 4,238
						Yami	8	0.04	0.04	720 (0; 2,131)	807 ± 807
						Ivatan	7	0.04	0.04	720 (0; 2,131)	807 ± 807
		
**E1a1a**	**14**	**1.07**	**0.33**	**8,423 (3,331; 13,515)**	**7,237 ± 2,232**	**Total**	**136**	**0.42**	**0.12**	**7,900 (3,471; 12,328)**	**8,476 **± 2,422
						Ivatan	6	0.01	0.01	205 (0; 608)	202 ± 202
		
**E2a**	**15**	**1.07**	**0.34**	**8,423 (3,177; 13,669)**	**7,237 ± 2,299**	**Total**	**20**	**0.65**	**0.32**	**12,251 (428; 24,074)**	**13,117 **± 6,458
						Ivatan	1	NA	NA	NA	NA
		
**E2b1**	**6**	**0.5**	**0.29**	**3,936 (0; 8,410)**	**3,382 ± 1,961**	**Total**	**44**	**0.16**	**0.12**	**2,998 (0; 7,353)**	**3,229 **± 2,422
						Yami	5	0.03	0.03	496 (0; 1,467)	605 ± 605
						Ivatan	1	NA	NA	NA	NA
		
**E2b2**	**2**	**NA**	**NA**	**NA**	**NA**	**Total**	**8**	**0.38**	**0.18**	**7,069 (535; 13,603)**	**7,668 **± 3,632
						Ivatan	7	0.14	0.14	2,689 (0; 7,960)	2,825 ± 2,825
		
F1a1d*	**10**	**1.4**	**0.79**	**11,021 (0; 23,210)**	**9,469 ± 5,343**	**Total**	**40**	**0.33**	**0.17**	**6,127 (0; 12,258)**	**6,659 **± 3,431
						Yami	18	0.07	0.05	1,261 (0; 3,000)	1,413 ± 1,009
		
**F1a3**	**7**	**0.86**	**0.35**	**6,770 (1,370; 12,170)**	**5,817 ± 2,367**	**Total**	**41**	**0.49**	**0.17**	**9,189 (3,076; 15,302)**	**9,888 **± 3,431
						Ivatan	3	NA	NA	NA	NA
		
**F1a4**	**5**	**2.4**	**1.02**	**18,893 (3,155; 34,631)**	**16,233 ± 6,899**	**Total**	**41**	**0.39**	**0.16**	**7,359 (1,445; 13,272)**	**7,870 **± 3,229
						Ivatan	9	0.03	0.03	654 (0; 1,936)	605 ± 605
		
**M7b3**	**6**	**1.5**	**0.5**	**11,808 (4,093; 19,523)**	**10,146 ± 3,382**	**Total**	**108**	**0.61**	**0.28**	**11,467 (1,088; 21,846)**	**12,310 **± 5,650
						Yami	8	0.01	0.01	271 (0; 803)	202 ± 202
		
M7b4*	**5**	**2.6**	**1**	**20,467 (5,038; 35,896)**	**17,586 ± 6,764**	**Total**	**25**	**0.84**	**0.47**	**15,828 (0; 33,125)**	**16,951 **± 9,485
						Ivatan	1	NA	NA	NA	NA
		
**M7c3**	**22**	**1.36**	**0.27**	**10,706 (6,540; 14,872)**	**9,199 ± 1,826**	**Total**	**138**	**1.38**	**0.39**	**25,914 (11,638; 40,190)**	**27,848 **± 7,870
						Yami	9	0.1	0.1	1,886 (0; 5,584)	2,018 ± 2,018
		
M7c3c2*	**2**	**2**	**1.41**	**15,744 (0; 37,499)**	**13,528 ± 9,537**	**Total**	**22**	**0.64**	**0.59**	**11,991 (0; 33,883)**	**12,915 **± 11,906
						Ivatan	1	NA	NA	NA	NA
		
N9a10*	**5**	**2.2**	**1.18**	**17,318 (0; 35,525)**	**14,880 ± 7,981**	**Total**	**17**	**0.24**	**0.19**	**4,436 (0; 11,299)**	**4,843 **± 3,834
						Ivatan	3	NA	NA	NA	NA

* Haplogroups not described in van Oven 2009: F1a1d, M7b4, and N9a10 (see text).

^# ^Molecular dating of mtDNA sequences for coding region synonymous mutations was obtained using a rate of one synonymous mutations per 7,884 years [53] or per 6,764 years [51].

^$ ^Molecular dating of mtDNA HVS-1 sequences (n > = 5) was obtained using a rate of one transition per 19,171 years when Rho method was applied according to [53], or per 20,180 years according to [52,64].

NA: Not applicable.

Note: Inferences made from such dates warrant caution as they have considerable uncertainty and may be inaccurate [33].

^&^All lineages were identical.

1) Firstly, haplogroups shared between Yami and Ivatan (B4a1a (including B4a1a4), B4a2a and B4c1b2) showed age between ~800 to 1,600 YBP (95% CI; 0 to 4,600 years) as estimated by HVS-1 polymorphism. Compared to the archaeological estimates of settlement [5,7], our observation suggested that a permanent settlement must have post-dated the first traces of human activities observed on Orchid or Batanes islands (A caution is noted because there is an estimate overlap between the 95% confidence interval (CI) and the archeological estimate).

2) Except for the Yami haplogroups M7c3c and E2b1 which had only one representative in Ivatan, no other non-B4 haplogroups were shared between Yami and Ivatan. The two groups of islanders were clearly differentiated by two patterns, haplogroups F1a1d and M7b3 in Yami and haplogroups E1a1a, E2a, E2b2, F1a3, F1a4, M7b4, and N9a10 in Ivatan. While F1a1d and M7b4 have been reported in MSEA [34] (Figure 2, Table 1), all other haplogroups have only been seen in ISEA or among TwA. This suggested that the only maternal influence (via Taiwan) from MSEA was limited to F1a1d and M7b4, and that most Yami or Ivatan could trace their ancestry to either ISEA (i.e. B4a1a4, E2a, E2b2, F1a3 and F1a4) or to Taiwan (i.e. B4a2a, E2b1 and M7b3a). Further, the largest molecular variation among these haplogroups within the Yami, gave a 95% confidence interval on an age estimate that is within 3,000 YBP (Table 2). This again supports a more recent stage of permanent settlement on Orchid Island compared to the archaeological estimate of 4,000 YBP.

In summary, while Yami (with all haplogroups except B4a1a4 and B4c1b2) showed a stronger relationship with Taiwan, the Ivatan showed a closer affinity to the Philippines or Taiwan than to Yami. If not considering genetic drift, this pattern indicates bypassing of the Batanes Islands in the early stage of "Out of Taiwan", and later colonization of the Batanes from Luzon. The evidence of bidirectional maternal gene flow between the two islands was inferred from a time of settlement not exceeding 3,000 YBP.

### Y chromosome

The frequencies of Y-chromosome single nucleotide polymorphisms (Y-SNP) haplogroups are shown in table 3. As previously reported for Taiwan and ISEA [35-37], O1-M119 and O2-P31 were the most common haplogroups among Yami, but O2-P31 was not seen in Ivatan and not so common in the Philippines. Interestingly, macro haplogroups K and NO*, indicators of Paleolithic traces for ISEA (9% to 46%) and the Philippines (0% to 6%), were not seen in the Orchid or Batanes Islanders [38]. Also, except for the presence of one O3a4*-GPS002611 lineage in Yami and one O1a1*-P203x lineages in Ivatan (Table 3), Y-SNP sharing between Yami and Ivatan was restricted to haplogroup O1a*-M119x.

**Table 3:** Frequencies of Ivatan and Yami NRY haplogroups and corresponding frequencies in nearby populations

**Haplogroups**	**China**	**Taiwan**										**Philippines**	
			
	**Fujian (Han)** **(53)**	**Taiwanese (Han)** **(94)**	**Aborigines**									**Batanes**	**Pooled populations**
				
			**Atayal** **(52)**	**Saisiat** **(24)**	**Bunun** **(56)**	**Tsou** **(41)**	**Ami** **(39)**	**Paiwan** **(25)**	**Puyuma** **(23)**	**Rukai** **(29)**	**Yami** **(30)**	**Ivatan** **(24)**	**Philippines**^**# **^ **(122)**
		
O1a*-M119x		1.1	7.7			4.9		24.0	13.0	6.9	33.3	41.6	12.3
O1a1*-P203x	22.6	12.8	90.4	87.5		90.2	41.0	40.0	47.8	69.0	50.0	4.2	15.6
O1a2-M110		1.1	1.9	4.2	60.7	4.9	17.9	28.0	21.7	24.1		16.7	10.6
O2a*-M95x	5.7	6.4									10.0		
O2a1a-PK4		2.1			37.5						3.3		4.1
O3a3*-P201x	3.8	4.3					35.9	4.0	17.4			12.5	19.7
O3a4*-GSP002611, xP103	26.4	16.0									3.3	25.0	0.8
		
Haplogroup diversity ± SD	0.84	0.88	0.18	0.23	0.49	0.18	0.67	0.70	0.68	0.46	0.63	0.72	0.89
		
	± 0.017	± 0.012	± 0.048	± 0.079	± 0.024	± 0.056	± 0.025	± 0.029	± 0.044	± 0.060	± 0.039	± 0.036	± 0.008
		
Number of shared haplogroups with Yami & Ivatan	3 & 3	5 & 5	2 & 3	1 & 2	1 & 1	2 & 3	1 & 2	2 & 4	2 & 4	2 & 3	NA & 3	3 & NA	4 & 5
		
Total Frequency	58.5	43.6	100.0	91.7	98.2	100.0	94.8	96.0	100.0	100.0	100.0	100.0	63.2

Except for Yami and Ivatan all data is obtained from (Trejaut et al. material in preparation). Taiwanese includes Minnan and Hakka (these groups are not differentiable using the exact test of population differentiation test (Arlequin Package [55]).

# No Ivatan included; NA = not applicable

x after an SNP marker indicates this SNP was ancestral (no other derived SNPs were seen).

x before an SNP indicate this SNP was not present.

The median joining (MJ) networks were constructed using Yami and Ivatan polymorphisms obtained from 16 Y-STR loci in each Y-SNP haplogroup (O1a*-M119, O1a1*-P203, O1a2-M110, O2a*-M95, O2a1a-PK4, O3a3*-P201 and O3a4*-GSP002611) (Additional file 3). Only five distinct O1a*-M119 Yami Y-STR haplotypes were found (Additional file 1). These haplotypes were not shared between the two islands, suggesting drift, sampling bias or an absence of recent paternal gene flow between Yami and Ivatan. No clusters of Yami or Ivatan Y-STR lineages were found with TwA, and Indonesia (Additional file 3). Nonetheless the haplogroup O1a1*-P203 network showed some relationships among Philippine Y-STR lineages and five Yami individuals from Iraralai (including three from family 48, one individual from families 44 and one from family 45) suggesting the peoples of Orchid island and the Philippines are related.

On the other hand, age estimates according to molecular variation [39] in Y-STR clusters suggested possible local founding events not exceeding 3,230 YBP (± 1,400 years) for Yamis (Additional file 4) and 3,300 YBP (± 1,430 years) for Ivatans (data not shown).

A population phylogenetic tree was constructed using Y-STR *F*_st _distances between all the groups in our dataset and the other populations in SEA [40-44]. Yamis, Ivatans, Amis and Filipinos shared a close paternal relationship; this result agreed with the phylogenetic pattern from mtDNA studies (Additional file 2). Nonetheless, these ethnic groups also showed Y-STR affinity to the Southern Taiwan Aboriginal tribes (Paiwan, Rukai and Puyuma) probably indicating a greater inter-island movement of men than women. We also noticed that the few shared haplotypes between Yami and MSEA belong to the haplogroups O1a*-M119, O2a*-M95 and O2a1a-PK4 (Malaysia, Thailand, Southwest China, and Malagasy). Similarly, some haplotypes shared by Ivatan and Malaysia belong to haplogroups O1a2-M110 and O3a3*-P201.

### Analysis of molecular variance (AMOVA)

Using the information shown in Additional file 1, the paternal and maternal lineages among Yamis were regrouped according to village of paternal and maternal origins. Analysis of molecular variance (AMOVA) [45] between maternal lineages and their village of origin (Table 4) did not show much differences among villages (*F*_st_= 0.0055; P > 0.05) indicating that mtDNA lineages were distributed randomly throughout Orchid Island among women. On the contrary, the Y-STR paternal variation among villages varied significantly (*F*_st_= 0.17835; P < 0.0001) which suggest a sedentary life of the Yami men.

**Table 4:** AMOVA result of paternal and maternal lineages segregation by village in Yami

	Paternal Lineages (Y-STR)		Maternal Lineages (mtDNA HVS-1)	
	**Variance**	***F***_**st**_	**Variance**	***F***_**st**_

Between village variation	0.0895	0.17835*	0.0206	0.0055
Within village variation	0.4123		3.7015	
Total	0.50184		3.72205	

*P < 0.0001				

### Phylogenetic and Genealogy

**Figure 3:**
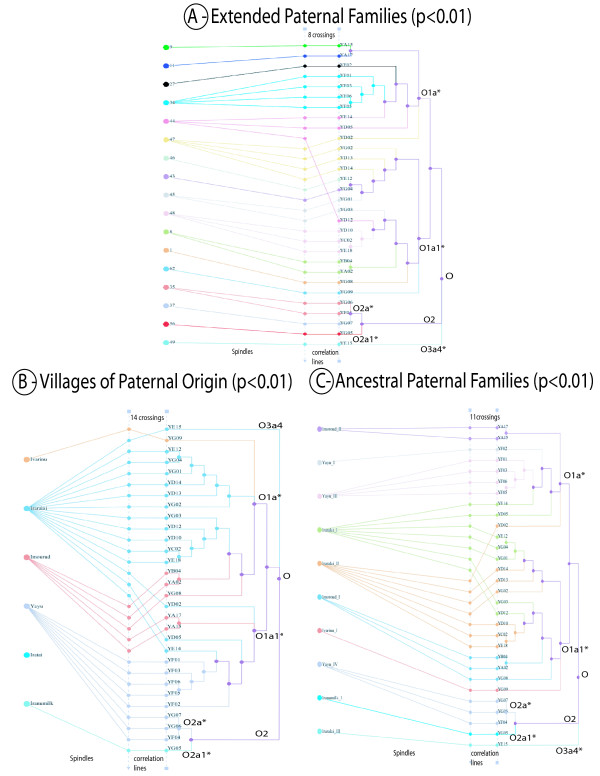
**Concordance between Yami NRY phylogenetic diversity (Y-SNP and Y-STR) and the genealogy survey of Wei and Liu (1962)**. Phylogenic tree of Y-SNP and Y-STR diversity. Each leaves (or one individual) represent a nuclear family. According to Wei and Liu conclusions (1962) [22], extended paternal families (left numbers in diagram C) and their ancestral families (Village + Roman numerals in diagram B) are not shared between villages. Quantitative visualization of the Wei and Liu relationship with NRY phylogenetic is done using the GenGIS program [46]. Each axis of categories on the left of diagrams A, B or C (i.e. Villages, Ancestral or extended families) have been ranked to introspect the Wei and Liu statement and represent the least number of crossings of correlation lines between the left axis and the leaves of the NRY Phylogram. The fit of each ordered genetic lay out to the genealogy of Wei and Liu was tested using a Monte Carlo permutation test on the leaf nodes. The fraction of crossings lesser than those shown in the figure (A = 14, B = 11 and C = 8) represent the P values. The P values were all < 0.01 [46] (see also Additional file 4) and indicate that concordance between the NRY phylogeny and the Wei and Liu paternal genealogy is not random. A - Villages of paternal origin. The spindles from villages represent the NRY distribution throughout Orchid Island. B - Ancestral paternal families' correspondences to the NRY phylogeny. Crossing correlation lines are all restricted to the Iraralai village indicating a few discrepancies between NRY Phylogeny and the Wei and Liu genealogy. C - Extended paternal families. Families 43 to 49 belong to the Iraralai village. Three families, 44, 45 and 47 have members belonging to different NRY subclades. Reiterating B, this pattern indicates erroneous Wei and Liu survey information or departure from a patrilocal way of life among Iraralai families but does not destroy the "one family-one village" relationship observed by Wei and Liu among Yami.

In Figure 3 (and Additional file 4), the ancestral and extended families in each village [22] were compared with the Yami NRY most parsimonious tree constructed from our Y-SNP and Y-STR results. Each Yami individual in the figures represents one nuclear family. The relationship between villages, ancestral and extended families (Left lay out of Figure 3A, B and 3C) have been arranged to represent the Wei and Liu model, in which "the Yamis are a patrilocal society where families and their ancestry are village specific" [22]. Accordingly, the correlation among extended families should extend to the correlation among most parsimonious tree and the lay outs (Figure 3). Deviations from this relationship would create crossings among the correlations lines. Quantitative visualization of the Wei and Liu relationship was constructed with the GenGIS software [46]. Fitting of the ordered lay outs to the corresponding phylogeny was tested using a Monte Carlo permutation test of the leaf nodes. The P values indicated that the fraction of crossings were lesser than what was set in the figure out of 1000 permutations [46] (Additional file 4). All P values (Figure 3A, B and 3C) were < 0.01 suggesting that the model used to represent the Wei and Liu hypothesis produced a significant number of correlation lines.

## Discussion

### Genetic relationship between Yamis and Ivatans

Substantial trading among the regions of MSEA, Taiwan and ISEA dated back to ~4,000 YBP was described in the literature indicating that all the islanders, including Yami, Ivatan and coastal dwellers from the China Sea, used advanced navigation techniques to sail forth and back among islands. Such findings were inferred by:

1. Artifacts found in Orchid Islands and Batanes that were dated back to the "Fine Corded Ware Culture" of Taiwan around ~4,000 YBP [5,7];

2. Jade trading among the Philippines, East Malaysia, southern Vietnam, Orchid Island, Batanes, and Thailand, that occurred between 2600 to 1500 YBP [8];

3. The presence of Y haplogroups O1a2 and O2a in Madagascar suggesting an establishment associated with the Austronesian expansion or people coming from Southeast Asians during 1,500 to 2,000 YBP [43,47];

4. Yami and Ivatan linguistically connected to the Western Malayo-Polynesian branch of Austronesian in ISEA [2].

In this study the matrilineal and patrilineal relationship between Yami, Ivatan, Taiwan Aborigines, the Philippine people, and other populations from the mainland and island Southeast Asia, were analyzed. Our goals were first test if there was a northward gene flow from the Philippines to Taiwan, and second to compare the Y chromosome data for the Yamis with paternal genealogy report by Wei and Liu (1962).

20% of the mtDNA haplogroups shared between Yami and Ivatan included B4a1a4, B4a2a, B4c1b2, E2b1, and M7c3c. The sharing of Y-SNP was higher (40.8%) and included haplogroup O1a*-M119, O1a1*-P203 and O3a4*-GSP002611. Lin et al. (manuscript in preparation) observed sharing between Taiwanese Han and TwA (23% for mtDNA haplogroups and 42% for Y-SNP). This increased Y-SNP contribution could reflect a sex biased social behavior. Alternatively, it could be associated with the slower mutation rate of the Y-SNP polymorphism that results in lower haplogroup diversity. However, using mtDNA (HVS-1 and relevant coding region information), Y-STR polymorphism and the Y-SNP diversity, no such disproportion of haplotype sharing was seen between Yami and Ivatan (mtDNA: 8%; Y-STR: 7%). The mtDNA haplogroup B4a1a4 defined by np 4025 (Yami 15%, Ivatan 4% and Philippines 1%) was the only representative one of the B4a1a clade in Yami. Its complete absence in Taiwan Aborigines and higher diversity in Filipinos suggests a northward gene flow from the Philippines within 3,000 years (Table 2). The two distinct branches of B4a1a4 seen in Yami and Ivatans (Figure 2) indicated that the islanders must have remained in isolation since settlement. Further, the total number of mtDNA haplogroups (Table 1) observed in Yami and Ivatan (7 and 15 respectively) were relatively small in comparison to that in Taiwanese Han and the Filipinos (77 and 43) indicating isolation and a small number of initial founders on the islands. This indication of isolation of the Yamis becomes plausible as only ten mtDNA haplotypes with frequencies ranging from 6 to 24% were sufficient to represent all the seven Yami mtDNA haplogroups. Alternatively, poor sampling, small population size on the small island, and genetic drift may all have influenced the genetic profiles observed [6].

All the Yami and Ivatan Y-SNP haplogroups belonged to the subgroups of macro haplogroup O which is seen throughout the MSEA and ISEA. The frequency of haplogroup O3 is high in Northern and Central Asia, whereas that of haplogroup O2 in south Asia and MSEA, and that of haplogroup O1 are being mostly distributed throughout ISEA [37,48,49]. The Y-SNP haplogroups seen in Yamis or Ivatans (subgroups of O1a, O2a, and O3a) also appear in MSEA and together represented a possible minimal haplogroup sharing of 26% between MSEA and either Yami or Ivatan. Nonetheless, a distinct contribution from MSEA to the islands was difficult to ascertain based of Y-SNP polymorphism alone. A matrilineal influence from MSEA was also indicated by the presence of the mtDNA haplogroups B4c1b2, F1a1d or M7b4 which determines a matrilineal contribution of 6% of the Yamis and of 14% with Ivatans. Many other mtDNA haplogroups seen in Taiwan and ISEA/Philippines suggest a direct gene flow from these locations to Batanes and/or to Orchid Island. For example by comparing haplogroup frequency and gene diversity, haplogroups B4a2a, and E2b1 (and to a lesser extent F1a1d and N9a10) suggested a gene flow from Taiwan, and haplogroup B4a1a4, B5b1, E2a and E2b2 suggested a gene flow from the Philippines. In general, Yami and Ivatan had stronger affinity with their closest larger neighbor.

Our mtDNA phylogenetic tree (Additional file 2) puts Yami and Amis in the same cluster as Ivatans and the Philippines. Except for the Amis, this clustering followed the same pattern as described by Ross (2005) indicating separate sub-branches of Batanic languages for Yamis and Ivatans both of which belong to the Western Malayo-Polynesian branch of the Austronesian language family, dated back to 2,500 YBP [2,50]. Also, age estimates from molecular variation of mtDNA haplogroup B4a1a4 and of Y chromosome O1a*-M119 in Yami and Ivatan indicated and overlap in the dating ranges (95% CI for mtDNA ranging from 0 to 3,000 YBP, and SE for Y-STR ranging from 750 to 3,230 YBP) (Table 2 and Additional file 4). The strong genetic affinity between Yamis and Taiwan Aborigines and the lack of genetic flow between Yamis and Ivatans (Additional file 3) led us to hypothesize that a language shift from Formosan to Malayo-Polynesian may have occurred among Yami. The language shift might not be associated with the gene flow from the Philippines but might have resulted from linguistic diffusion that was initiated by trading of jade or other goods in the region [8].

### The formation of Yami and Ivatan - time and people

Molecular Dating with the Rho Statistic [51-53] of mtDNA clades (Table 2) and/or Y-STR clusters (Additional file 4) of Yamis and Ivatans rarely exceeded ~2,000 years (SE 750 to 3,230) which differs from the archeological estimate of 4,000 years [5,7]. Thus the extant populations on these islands most likely represent a more recent family line of immigrants.

Interestingly, none of the mtDNA and Y chromosome haplogroups seen in Yami or Ivatan suggested a relationship with the eastern Melanesian populations where mtDNA haplogroups P and Q, and Y-SNP haplogroups D, C, F and K are prominent [27,35,54]. A few mtDNA haplogroups among Yamis or Ivatans originated either in Taiwan (B4a2a, E2b1, F1a1d, and N9a10) or the Philippines (B4a1a4, E2a, E2b2 and B5b1). All the remaining haplogroups were commonly seen in Taiwan Aborigines and Filipinos. The data suggested a bidirectional gene flow and support the "Viaduct model" proposed by [27].

### Yami Paternal genealogy and Phylogenetic diversity

While the Y-SNPs haplogroups were heterogeneously distributed throughout Orchid Island (Figure 3B and Additional file 4), only one Y-STR lineage (represented by YF02 and YE14) was seen in two different villages (Additional file 4). Nonetheless, an AMOVA test using Y-STR lineages distribution among villages (Table 4) confirmed the patrilineal heterogeneity (P < 0.0001) throughout Orchid Island. On the contrary, the AMOVA test conducted by mtDNA lineages did not show significant matrilineal genetic variation within or among villages, indicating that the maternal genetic ancestry was homogeneously distributed throughout the island, and that male gene flow rarely occurred. This observation was supported by the anthropological study of Wei and Liu [22] and of Yu-mei Chen (private communication) who observed that intermarriage between villages were common for women from Iranumilk, Imourud and Ivarinu villages. Further the mtDNA analysis using an exact pairwise population differentiation test [55] did not show significant differences among the three villages (Iranumilk, Imourud and Ivarinu) and other villages on Orchid Island (data not shown).

We also investigated the Yami oral history which claims that people from Iraralai, Yayu, and Ivarinu had close relationships with the Ivatans (Yu-mei Chen private communication). In Additional files 3 and 4, two O1a*-M119 nuclear Yami families showed clustering with Ivatans (YD05 and YD12 from the extended families 44 and 47). No such relationships were found with Taiwan Aborigines or other region of MSEA. Another strong relationship was seen in the O1a1*-P203 network (Additional file 3) between YD13 (from family 46) and one Y-STR lineage carried by two Filipinos. Interestingly, our genetic data supported the oral history reported by (Yu-mei Chen private communication). We also investigated two other folk tales of Yami, one related to children adoption and the other related to people seeking refuge in another village. If child adoption indeed took place, this can be inferred from the correlation profile of the Iraralai families 44, 45 and 47 each having some family members in different genetic subclades (Additional file 4). Our NRY data were unable to support if the people from Imourud had migrated to Iraralai after a major flood in the island [6].

## Conclusions

A close genetic relationship between Yamis and Ivatans was hypothesized by linguistic studies, since both groups of islanders belong to the Batanic sub-branches of the Malayo Polynesian language group found in the ISEA. Accordingly, such a relationship would indicate a northward migration from the Philippines via Batanes archipelago and Orchid Island toward Taiwan. Our study, using Y-SNP and mtDNA polymorphism at the macro haplogroup level, showed that a strong affinity between the Yamis and Ivatans was resulted from gene flow between Taiwan and Philippines. Each island population showed a higher affinity with the closest main island (i.e., Yami with Taiwan, or Ivatan with Philippines) than with each other. This suggests an early isolation of the population and little intermarriage among the islands. Only few traces of gene flow were found between Yami and Ivatan or between Yami and Philippines. The gene flow appear independent from the cultural development, suggesting that trading had small impacts on genetic exchanges but must have resulted the linguistic affinity observed today among Yami, Ivatan and Philippines.

The age estimates of the mtDNA or Y-STRs variations suggested settlements on the islands dated back to ~3,000 YBP. However, the archeological artifacts found on Orchid Island and Batanes were associated with the "Out of Taiwan" hypothesis, indicating a southward migration from Taiwan and an earlier settlement on the islands that might be 4,000 YBP. These conflicting observations suggested that our sampling may have been too small to reveal sufficient or significant markers that can support a unique southward gene flow.

In Additional file 5 we propose three separate scenarios [2]. Briefly, scenarios 1 and 2 were proposed by Ross [2]. They correspond to the "Out of Taiwan" hypothesis (scenario 1, Additional file 5) and to a northward migration from Luzon to the Batanes archipelago (scenario 2, Additional file 5).

Ideally, any scenarios should consider variation due to drift, founder effect and admixture. Although the Out of Taiwan model [10] allows for some micro-spatial interactions, these conditions are ignored in a linguistic based model. The simple stepping stones of Neolithic dispersal represented by scenario 1 (Additional file 5) is not sufficient to associate with the complexity of genetic patterns observed in this study. We described that very little Y-STR sharing between Yami and Ivatan was seen (Additional file 3). Their mtDNA patterns/profiles was also very distinct. In general, the mtDNA haplogroups with high frequencies in one population was very low in the other population, but the mtDNA haplogroups were frequently matched among closest populations. Such variation could also be expected from a strong genetic drift (as indicated by the Tajima's D value (Table 1)). Scenario 3 (Additional file 5) seems to fit well with the mtDNA and Y-SNP data. It also evokes a much reticulated network of cultural relationships, and suggests (as for scenario 2) the possibility of northward Malayo-Polynesian language diffusion from Luzon (or from the Batanes Archipelago). While these hypotheses require further simulation testing, we propose that the extant genetic relationship observed between Yamis and Ivatans was resulted from complex events that occurred during the period of the Out of Taiwan and the subsequent trading between Taiwan and Luzon. Linguistic diffusion from Philippines may have also affected these events.

Finally, our diversity analysis of NRY Polymorphism diversity showed major concordance with the Wei and Liu paternal genealogies. Such ethnographic study of kinship provided insights to the complex and uncertain ways of how ideas of family ancestry, culture and linguistic contributed toward the formation of the Yami group identities, and how genetic revealed or confirmed their descent and their origins. Although the paternal relationships among the Yami groups determined by the survey of Wei and Liu covered only a few generations, it contributed clearly toward the groups self perception of their identity. However, these notions of relatedness were complicated by the accumulation of too much information, such as the complex and deeply rooted one brought upon by genetics. We showed how knowledge of ancestry, when combined with history, social relationships, genealogy and the use of several genetic systems, can be put to work to determine the idea of tribally pure lines of descent within families.

Despite the complex and ambivalent ways in which people perceive the cultural, biological and genetic constitution of ethnic identities, rapid social changes, frequent risk of ethnic group dilutions or their disappearance, make it an urgent requisite to obtain additional data from all minority groups, such as the Yami and Ivatan, to record more accurate extant profiles, and finally to favor multidisciplinary approaches.

## Methods

Seventy-nine unrelated Yami from Orchid Island (30 men and 49 women) were asked to participate in the study. All individuals provided their name, birthplace, the name of their parents and the village their parents came from. Among the 79 individuals, 12 mothers were from Imourud, 33 from Iraralai, 11 from Yayu, ten from Iratai, eight from Iranmilk, and five from Ivarinu (Figure 1). Among the 30 men, five were born in Imourud, 15 in Iraralai, eight in Yayu, one in Iranmilk, and one in Ivarinu (Additional file 1). Using subject's name, parents' names, and birthplace information, each Yami male individual was traced back to one of the extended families described in the Wei and Liu 's genealogy [22]. Since Wei and Liu's genealogy was based on patrilineality, only the Y chromosome phylogeny (Y-SNP and Y-STR) was used for comparison between the genealogy and genetics.

To analyze the relationship between the Yami and Ivatan, 50 unrelated Ivatan individuals (24 men and 26 women) were recruited from Itbayat, an island of the Batanes archipelago belonging to the Philippines (Figure 1).

All participants in this study gave informed consent to the study for collection of blood samples and DNA analysis. The project was approved by the ethics committee of Mackay Memorial Hospital, the Taiwan Health Department and the Philippines government.

To analyze the polymorphism of mtDNA and Y chromosome, DNA was extracted from 500 μl of buffy coat from each blood sample using the QIAmp DNA kit (QIAmp^® ^DNA Blood Mini kit from Qiagen inc. Taiwan). The non-recombining region of the Y chromosome (NRY) was determined using 70 single nucleotide polymorphisms (SNP) and 16 short tandem repeats (STRs). For mtDNA typing, control region HVS-1 [56], nucleotide positions (nps) of coding region fragments 8000 to 9000, 9800 to 10900 and 14000 to 15000 were sequenced using the method described in our previous publications [25,27]. When relevant to the study, complete mtDNA genome sequencing was carried out [25]. Briefly, 24 fragments of mtDNA were amplified and sequenced in both directions [25,57]. Haplogroup assignments were done according to the "Phylotree" criterion [26] available at http://www.phylotree.org using the combination of the HVS-1 sequence, partial sequencing of the coding region, and other relevant diagnostic variants of the coding region obtained by restriction fragment length polymorphism (RFLP) [25,27]. In addition, the presence of np 4025 indicating locally named mtDNA haplogroup B4a1a4 (Figure 2) was determined by sequence specific polymorphism (SSP) using forward primer 3999-4025 (5'TATTA TAATA AACAC CCTCA CCACT AT3'), and reverse primer 4049-4025 (5'TCATA TGTTG TTCCT ACCAA GATTG3') as internal primers of fragment 6 described by Rieder [25,57].

Y chromosome polymorphisms were ascertained using a hierarchical stepwise approach. For this, relevant SNPs were determined using direct sequencing of amplicons obtained from specific primer pairs as described in the Y Chromosome Consortium 2002 [58-60]. In brief, DNA samples were initially tested for super haplogroup O markers. Since all Yami and Ivatan samples were found to belong to this haplogroup, specific down stream markers of haplogroup O were then determined using more restricted primers [58]; [37]. Y STRs were subsequently determined in all individuals using 16 STRs (AmpFlSTR^® ^Y filer^® ^PCR Amplification Kit from Applied Biosystems, Taiwan).

### Data analysis

Frequencies of haplogroups among populations were obtained by direct counting (Table 1 and 3). On the basis of haplogroups frequency, mtDNA and Y-STRs distances matrices were obtained using *F*_st _distances after 10,000 permutations and a 0.05 significance level (ARLEQUIN package 3.1) [55]. Population phylogenetic trees were constructed using the neighbor-joining (NJ) method of (Saitou and Nei 1987) implemented in the Phylip package [61]. Test of neutrality, Tajima's D value (1989) [62], was calculated with DnaSP Sequence Polymorphism software package [63].

Specific *F*_st _indices to measure the variance of paternal or maternal lineages within and between villages was obtained from AMOVA using ARLEQUIN package 3.1 [55]. Ages of molecular variation for mtDNA were inferred using the ρ method for complete sequencing and HVS-1 data [51-53], using a rate of one synonymous transition per 7,884 years (bps 590-15990) and one transition per 19,171 years (bps 16090-16365) for the Soares method, or 6764 years and 20180 for Kivisild and Foster and Saillard methods respectively [51-53,64]. Y chromosome dates were estimated using Y-STR data in the background of their respective SNP haplogroups using the ρ statistic with an average mutation rate of 6.9 × 10^-4 ^± 5.7 × 10^-4 ^per locus per 25 years [39]. Generation length, bottlenecks, founder events and population size dynamics, geography are confounding factors that may cause unexpected variations of rho and warrant caution to inferences made from molecular variation [33].

Y-STR median joining networks were constructed using Network software 4.5.1.0 [65]. Finally, a Yami NRY phylogenetic tree was constructed using Y-SNP and Y-STR patterns in the background of each Y-SNP haplogroups, O1a*-M119, O1a1*-P203, O2a*-M95, O2a1a-PK4 and O3a4*-GSP002611 respectively (Additional file 3) [66,67]. Correlation between the village restricted paternal genealogy of Wei and Liu [22] and the leaves of the NRY Phylogeny was analyzed and visualized with the GenGIS package [68]. Accordingly, extended families, villages and ancestral families (Figure 3A, B and 3C respectively) were first separately laid out to obtain the minimum number of correlation lines crossings between the genealogic lay out and the leaves of the NRY phylogeny. A Monte Carlo permutation test was performed on the leaves of the Phylogenetic tree to assess if the fit was significantly better than random.

## Accession Numbers

The GenBank accession numbers http://www.ncbi.nlm.nih.gov/nucleotide/ for HVS-1 data in this article are as follows: HVS-1 (HM238219- HM238267). Complete sequence data: accession numbers HM238197- HM238218.

## List of abbreviations

TwA: Taiwan Aborigines; non TwA: non Taiwan Aborigines; YBP: years before present; MSEA: Mainland Southeast Asia; ISEA: Island Southeast Asia; NRY: non-recombining region of the Y chromosome; HVS-1: mitochondrial DNA hypervariable region 1; np: nucleotide position; SSP: Sequence Specific Polymorphism; SNP: Single Nucleotide Polymorphism; STR: Short Tandem Repeat.

## Authors' contributions

JHL and JAT wrote the paper. JHL and JAT performed population genetic analyses. ML, JHL and JAT conceived and designed the study. ML contributed DNA samples. JHL, JCY, ZSC and CLL performed sequence analysis. All authors read and approved the final manuscript.

## Supplementary Material

Additional file 1 **Sample information**. Click here for file

Additional file 2 **Phylogenetic tree of populations of Taiwan, ISEA and MSEA using mtDNA (top) haplogroup frequencies (*F*_st _distances) and Y-STR haplotypes frequencies (bottom)**. All mtDNA data information was obtained from the present study and from (Trejaut et al.; material in preparation). Y-STR data on Taiwan and ISEA was obtained from the present study and information for Mainland Southeast Asia populations was obtained from [39-43]. Click here for file

Additional file 3 **Y-STR networks of Yami, Ivatan and other populations of ISEA and MSEA**. Median-joining network for Taiwan, Southeast Asia and Island Southeast Asia of 16 Y-STR' variations within Haplogroup of O1, O2 and O3 (DYS19, DYS385a/b, DYS389I, DYS390, DYS390II, DYS391, DYS392, DYS393, DYS437, DY438, DYS439, DYS448, DYS456, DYS458, DYS635(YGATAC4), DYS635(YGATAH4). Circle areas are proportional to haplotype frequency and lines are the mutational differences between haplotypes. Click here for file

Additional file 4 **Concordance between Yami NRY phylogenetic diversity (Y-SNP and Y-STR) and Wei and Liu ethnographic study of kinship (1962)**. Villages are represented by boxes and center brackets between Roman numerals. Each family is represented by a single Y-STR lineage along the correlation lines. The Correlation were obtain with the GenGIS program [68]. Concordance between Yami NRY phylogenetic diversity (Y-SNP and Y-STR) and the genealogy survey of Wei and Liu (1962) [22]. Click here for file

Additional file 5 **Possible settlement scenarios of Orchid Island and the Batanes archipelago from Taiwan or the Philippines**. Scenario 1 is inspired from Ross linguistic study [2] and supports the "Out of Taiwan" model. The immediate ancestors of Proto Malayo-Polynesian speakers migrated out of Taiwan (~6,000 YBP to 4,000 YBP) to Orchid Island, the Batanes islands and Luzon, and developed languages specific to each regions (Figure 1). Scenario 2 is also inspired from Ross linguistic study [2]. In brief, the Proto Malayo-Polynesian origin is not located, but Northern Luzon is assumed to be a center of dispersion. As such, Orchid and Batanes islands could have been bypassed/ignored by the first migrants going from Taiwan to Northern Luzon (6,000 YBP to 4,000 YBP). Proto-Batanic languages would have developed during and after migrations from Luzon to the Batanes and Orchid islands (~3,000 YBP) where local languages later became more specific to Ivatan or Yami. Scenario 3 is based on genetics studies with first, a Bellwood-like expansion of people out of Taiwan ~4,000 years ago [10]. Secondly, Orchid and Batanes Islands could have been re-colonized from the south (as early as ~3,000 years ago, given the genetic estimates). Thirdly, later gene flow from Taiwan or Luzon would have affected the genetic profiles of people from Orchid or Batanes islands to look more like Taiwanese Aborigines or Filipinos respectively. Alternatively, the second stage could have been restricted to Ivatan who later extended their influence to Yami. This scheme is compatible with anthropological studies reporting that little to no external influence between Yami and Taiwan occurred from 1,500 YBP to 300 YBP [4]. The historically reported movement of people back and forth between Ivatan and Luzon during the 18th century typhoon and famine [23] most likely intensified Ivatan genetic affinity with Luzon and supports the last stage of this scenario. Click here for file

## References

[B1] Sanchez-Mazas A Poloni ES Jacques G Sagart L Laurent Sagart HLA genetic diversity and linguistic variation in East Asia. In"The Peopling of East Asia Putting Together Archaeology, Linguistics and Genetics" 2005 Centre Nationale de Recherche Scientique, France, Roger Blench, Overseas Development Institute, UK and Alicia Sanchez-Mazas, University of Geneva, Switzerland RoutledgeCurzon: RoutledgeCurzon 273 296

[B2] Ross M The Batanic Languages in Relation to the Early History of the Malayo-Polynesian Subgroup of Austronesian Journal of Austronesian Studies 2005 1 2 1 24

[B3] Blust R Subgrouping, circularity and extinction: some issues in Austronesian comparative linguistics Symp Ser Inst Linguist Acad Sinica 1999 1 31 94

[B4] Chen YM The formation of the Yami/Tao: an areal and historical perspective Formation and Reinvention of cultures and Ethnic groups among the Austronesians in Taiwan Research Group 2008 Institute of Ethnology, Academia Sinica: Academia Sinica

[B5] Tsang Ch On the origin of the Yami People of Lanyu as Viewed from Archaeological Data Journal of Austronesian Studies 2005 1 1 135 151

[B6] Chen YM Tai'Tong county history, Yami tribe section (in chinese) 2001 Tai'Tong county Government Taiwan

[B7] Bellwood P Dizon E The Batanes Archaeological Project and the "Out of Taiwan" Hypothesis for Austronesian Dispersal Journal of Austronesian Studies 2005 1 1 1 33

[B8] Hung H Iizukac Y Bellwood P Nguyene K Bellinaf B Silapanthg P Dizonh E Santiagoh R Datani I Mantonj J Ancient jades map 3,000 years of prehistoric exchange in Southeast Asia Proc Natl Acad Sci USA 2007 104 50 19745 19750 10.1073/pnas.0707304104 18048347 PMC2148369

[B9] Anderson A Crossing the Luzon Strait: Archaeological Chronology in the Batanes Islands, Philippines and the Regional Sequence of Neolithic Dispersal Journal of Austronesian Studies 2005 1 2 25 45

[B10] Bellwood P Dizon E Alicia Sanchez-Mazas RB, Malcolm D Austronesian cultural origins. Out of Taiwan, via the Batanes Islands, and onwards to western Polynesia Past Human Migrations in East Asia Matching archeology, linguisticx and genetics 2008 Ross, Ikia Peiros and Marie Lin. London and New York. 3-19: Routledge Taylor & Francis Group

[B11] Kano T Prehistoric and Ethnographic Studies of Southeast Asia I 1946 Tokyo: Yazima Publishing House

[B12] Kano T Bashic Channel and the cultural relationships between Taiwan and the Philippines Prehistoric and Ethnographic Studies of Southeast Asia I 1946 Tokyo: Yazima Publishing House

[B13] Kano T The interaction and disconnection between Kotosho and the Batanese Archipelago Prehistoric and Ethnographic Studies of Southeast Asia I 1946 Tokyo: Yazima Publishing House

[B14] Kano T Gold cultures of the Taiwan aborigines, the Philippines and Kotosho Journal of Anthropology 1941 56 465 478

[B15] Kano T The relationships between Kotosho and the Batanese Archipelago in terms of the nomenchature of fauna and flora Journal of Anthropology 1941 56 434 446

[B16] Kano T Jar burials found in Kotosho Journal of Anthropology 1941 56 117 135

[B17] Kano T Migration along the route from Orchid Island, Batanese Archipelago to the Philippines New Asia 1940 2 26 36

[B18] Utsushigawa Nea Genealogical Studies of the Taiwan Aborigines Department of Ethnography and Anthropology 1935 Taipei: Taipei Imperial University

[B19] Utsushigawa N Oral traditions and the relationships between the Yami of Kotosho and the Batanese Archipelago of the Philippines Southern Folklore 1931 1 15 37

[B20] Asai E Material culture and the relationship between Batan and the Yami Southern Folklore 1939 5 3/4 1 5

[B21] Kano, Tadao, Segawa, Kokichi An Illustrated Ethnography of Formosan Aborigines, Tokyo The Yami (revised edition) 1956 1 456

[B22] Wei HL Liu PH Social Structure of the Yami Botel Tobago 1962 Nankang, Taipei, Taiwan: Academia Sinica

[B23] Llorente AMM A Blending of Cultures: The Batanes 1686~1898 1983 XXXVIII Manila: Historical Conservation Society

[B24] Chu CC Lin M Nakajima F Lee HL Chang SL Juji T Tokunaga K Diversity of HLA among Taiwan's indigenous tribes and the Ivatans in the Philippines Tissue Antigens 2001 58 1 9 18 10.1034/j.1399-0039.2001.580102.x 11580850

[B25] Trejaut JA Kivisild T Loo JH Lee CL He CL Hsu CJ Li ZY Lin M Traces of archaic mitochondrial lineages persist in Austronesian speaking Formosan populations PloS 2005 3 8 1362 1372 10.1371/journal.pbio.0030247 PMC1166350 15984912

[B26] van Oven M Kayser M Updated comprehensive phylogenetic tree of global human mitochondrial DNA variation Hum Mutat 2009 30 2 E386 E394 10.1002/humu.20921 18853457

[B27] Tabbada KA Trejaut J Loo JH Chen YM Lin M Mirazon-Lahr M Kivisild T De Ungria MC Philippine mitochondrial DNA diversity: a populated viaduct between Taiwan and Indonesia? Mol Biol Evol 2010 27 1 21 31 10.1093/molbev/msp215 19755666

[B28] Hill C Soares P Mormina M Macaulay V Clarke D Blumbach PB Vizuete-Forster M Forster P Bulbeck D Oppenheimer S A mitochondrial stratigraphy for island southeast Asia Am J Hum Genet 2007 80 1 29 43 10.1086/510412 17160892 PMC1876738

[B29] Kong QP Bandelt HJ Sun C Yao YG Salas A Achilli A Wang CY Zhong L Zhu CL Wu SF Updating the East Asian mtDNA phylogeny: a prerequisite for the identification of pathogenic mutations Hum Mol Genet 2006 15 13 2076 2086 10.1093/hmg/ddl130 16714301

[B30] Oota H Kitano T Jin F Yuasa I Wang L Ueda S Saitou N Stoneking M Extreme mtDNA homogeneity in continental Asian populations Am J Phys Anthropol 2002 118 2 146 153 10.1002/ajpa.10056 12012367

[B31] Li H Cai X Winograd-Cort ER Wen B Cheng X Qin Z Liu W Liu Y Pan S Qian J Mitochondrial DNA diversity and population differentiation in southern East Asia Am J Phys Anthropol 2007 134 4 481 488 10.1002/ajpa.20690 17668442

[B32] Tsai LCL CY Lee JC Chang JG Linacre A Goodwin W Sequence polymorphism of mitochondrial D-loop DNA in the Taiwanese Han population Forensic Sci Int 2001 119 239 247 10.1016/S0379-0738(00)00439-4 11376990

[B33] Cox MP Accuracy of molecular dating with the rho statistic: deviations from coalescent expectations under a range of demographic models Hum Biol 2008 80 4 335 357 10.3378/1534-6617-80.4.335 19317593

[B34] Kong QP Yao YG Sun C Bandelt HJ Zhu CL Zhang YP Phylogeny of east Asian mitochondrial DNA lineages inferred from complete sequences Am J Hum Genet 2003 73 3 671 676 10.1086/377718 12870132 PMC1180693

[B35] Scheinfeldt L Friedlaender F Friedlaender J Latham K Koki G Karafet T Hammer M Lorenz J Unexpected NRY chromosome variation in Northern Island Melanesia Mol Biol Evol 2006 23 8 1628 1641 10.1093/molbev/msl028 16754639

[B36] Su B Jin L Underhill P Martinson J Saha N McGarvey ST Shriver MD Chu J Oefner P Chakraborty R Polynesian origins: insights from the Y chromosome Proc Natl Acad Sci USA 2000 97 15 8225 8228 10.1073/pnas.97.15.8225 10899994 PMC26928

[B37] Li H Wen B Chen SJ Su B Pramoonjago P Liu Y Pan S Qin Z Liu W Cheng X Paternal genetic affinity between Western Austronesians and Daic populations BMC Evol Biol 2008 8 146 10.1186/1471-2148-8-146 18482451 PMC2408594

[B38] Karafet TM Hallmark B Cox MP Sudoyo H Downey S Lansing JS Hammer MF Major East-West Division Underlies Y Chromosome Stratification Across Indonesia Mol Biol Evol 2010 20207712 10.1093/molbev/msq063

[B39] Zhivotovsky LA Underhill PA Cinnioglu C Kayser M Morar B Kivisild T Scozzari R Cruciani F Destro-Bisol G Spedini G The effective mutation rate at Y chromosome short tandem repeats, with application to human population-divergence time Am J Hum Genet 2004 74 1 50 61 10.1086/380911 14691732 PMC1181912

[B40] Chang YM Perumal R Keat PY Kuehn DL Haplotype diversity of 16 Y-chromosomal STRs in three main ethnic populations (Malays, Chinese and Indians) in Malaysia Forensic Sci Int 2007 167 1 70 76 10.1016/j.forsciint.2006.01.002 16457976

[B41] Chang YM Swaran Y Phoon YK Sothirasan K Sim HT Lim KB Kuehn D Haplotype diversity of 17 Y-chromosomal STRs in three native Sarawak populations (Iban, Bidayuh and Melanau) in East Malaysia Forensic Sci Int Genet 2009 3 3 e77 80 10.1016/j.fsigen.2008.07.007 19414156

[B42] Feng DL Liu CH Liang ZR Liu C Genetic polymorphism of 17 Y-STR loci in four minority populations in Guangxi of China Yi Chuan 2009 31 9 921 935 19819845 10.3724/sp.j.1005.2009.00921

[B43] Tofanelli S Bertoncini S Castri L Luiselli D Calafell F Donati G Paoli G On the origins and admixture of Malagasy: new evidence from high-resolution analyses of paternal and maternal lineages Mol Biol Evol 2009 26 9 2109 2124 10.1093/molbev/msp120 19535740

[B44] Zhu B Wu Y Shen C Yang T Deng Y Xun X Tian Y Yan J Li T Genetic analysis of 17 Y-chromosomal STRs haplotypes of Chinese Tibetan ethnic group residing in Qinghai province of China Forensic Sci Int 2008 175 2-3 238 243 10.1016/j.forsciint.2007.06.012 17659855

[B45] Excoffier L Smouse PE Quattro JM Analysis of molecular variance inferred from metric distances among DNA haplotypes: application to human mitochondrial DNA restriction data Genetics 1992 131 2 479 491 1644282 10.1093/genetics/131.2.479 PMC1205020

[B46] Parks DH Beiko RG Quantitative visualizations of hierarchically organized data in a geographic context Geoinformatics 2009 Fairfax, VA

[B47] Ricaut FX Razafindrazaka H Cox MP Dugoujon JM Guitard E Sambo C Mormina M Mirazon-Lahr M Ludes B Crubezy E A new deep branch of eurasian mtDNA macrohaplogroup M reveals additional complexity regarding the settlement of Madagascar BMC Genomics 2009 10 605 10.1186/1471-2164-10-605 20003445 PMC2808327

[B48] Shi H Dong YL Wen B Xiao CJ Underhill PA Shen PD Chakraborty R Jin L Su B Y-chromosome evidence of southern origin of the East Asian-specific haplogroup O3-M122 Am J Hum Genet 2005 77 3 408 419 10.1086/444436 16080116 PMC1226206

[B49] Kumar V Reddy AN Babu JP Rao TN Langstieh BT Thangaraj K Reddy AG Singh L Reddy BM Y-chromosome evidence suggests a common paternal heritage of Austro-Asiatic populations BMC Evol Biol 2007 7 47 10.1186/1471-2148-7-47 17389048 PMC1851701

[B50] Gray RD Drummond AJ Greenhill SJ Language phylogenies reveal expansion pulses and pauses in Pacific settlement Science 2009 323 5913 479 483 10.1126/science.1166858 19164742

[B51] Kivisild T Shen P Wall DP Do B Sung R Davis K Passarino G Underhill PA Scharfe C Torroni A The role of selection in the evolution of human mitochondrial genomes Genetics 2006 172 1 373 387 10.1534/genetics.105.043901 16172508 PMC1456165

[B52] Saillard J Forster P Lynnerup N Bandelt HJ Nørby S mtDNA variation among Greenland Eskimos: the edge of the Beringian expansion Am J Hum Genet 2000 67 718 726 10.1086/303038 10924403 PMC1287530

[B53] Soares P Ermini L Thomson N Mormina M Rito T Rohl A Salas A Oppenheimer S Macaulay V Richards MB Correcting for purifying selection: an improved human mitochondrial molecular clock Am J Hum Genet 2009 84 6 740 759 10.1016/j.ajhg.2009.05.001 19500773 PMC2694979

[B54] Capelli C Wilson JF Richards M Stumpf MP Gratrix F Oppenheimer S Underhill P Pascali VL Ko TM Goldstein DB A predominantly indigenous paternal heritage for the Austronesian-speaking peoples of insular Southeast Asia and Oceania Am J Hum Genet 2001 68 2 432 443 10.1086/318205 11170891 PMC1235276

[B55] Excoffier L Laval G Schneider S Arlequin ver. 3.0: An integrated software package for population genetics data analysis Evolutionary Bioinformatics Online 2005 1 47 50 19325852 PMC2658868

[B56] Wilson AC Polanskey D Butler J Dizinno J Replogle J Budowle B Extraction, PCR amplification and sequencing of mitochondrial DNA from human hair shafts. In: Book Biotechniques 1995 18 662 669 7598901

[B57] Rieder MJ Taylor SL Automating the identification of DNA variations using quality-based fluorescence re-sequencing: analysis of the human mitochondrial genome Nucleic Acids Res 1998 26 967 973 10.1093/nar/26.4.967 9461455 PMC147367

[B58] Karafet TM Mendez FL Meilerman MB Underhill PA Zegura SL Hammer MF New binary polymorphisms reshape and increase resolution of the human Y chromosomal haplogroup tree Genome Res 2008 18 5 830 838 10.1101/gr.7172008 18385274 PMC2336805

[B59] Underhill PA Shen P Lin AA Jin L Passarino G Yang WH Kauffman E Bonne-Tamir B Bertranpetit J Francalacci P Y chromosome sequence variation and the history of human populations Nat Genet 2000 26 3 358 361 10.1038/81685 11062480

[B60] YCC Y Chromosome Consortium, A nomenclature system for the tree of human Y-chromosomal binary haplogroups Genome Res 2002 12 2 339 348 10.1101/gr.217602 11827954 PMC155271

[B61] Felsenstein J Phylip; Phylogeny Inference Package Version 3.6(alpha3) edn. Seattle 2002

[B62] Tajima F Statistical method for testing the neutral mutation hypothesis by DNA polymorphism Genetics 1989 123 3 585 595 2513255 10.1093/genetics/123.3.585 PMC1203831

[B63] Rozas J DNA sequence polymorphism analysis using DnaSP Methods Mol Biol 2009 537 337 350 full_text 19378153 10.1007/978-1-59745-251-9_17

[B64] Forster P Harding R Torroni A Bandelt HJ Origin and evolution of Native American mtDNA variation: a reappraisal Am J Hum Genet 1996 59 935 945 8808611 PMC1914796

[B65] Bandelt H Forster P Rohl A Median-joining networks for inferring intraspecific phylogenies Mol Biol Evol 1999 16 37 48 10331250 10.1093/oxfordjournals.molbev.a026036

[B66] YCC A nomenclature system for the tree of human Y-chromosomal binary haplogroups Genome Res 2002 12 2 339 348 10.1101/gr.217602 11827954 PMC155271

[B67] Karafet TM Osipova LP Gubina MA Posukh OL Zegura SL Hammer MF High levels of Y-chromosome differentiation among native Siberian populations and the genetic signature of a boreal hunter-gatherer way of life Hum Biol 2002 74 6 761 789 10.1353/hub.2003.0006 12617488

[B68] Parks DH Porter M Churcher S Wang S Blouin C Whalley J Brooks S Beiko RG GenGIS: A geospatial information system for genomic data Genome Res 2009 19 10 1896 1904 10.1101/gr.095612.109 19635847 PMC2765287

[B69] Weng S Liou CW Lin TK Wei YW Lee CF Eng HL Chen SD Liu RT Chen JF Chen IY Chen MH Wang PW Association of mitochondrial deoxyribonucleic acid 16189 variant (T-C transition) with metabolic syndrome in Chinese adults The Journal of Clinical Endocrinology & Metabolism 2005 90 5037 5040 10.1210/jc.2005-0227 15972579

