# Supplementary figures and images for: Genetic affinities between the Yami tribe people of Orchid Island and the Philippine Islanders of the Batanes archipelago
Source: BMC Genet. 2011 Jan 31;12:21. doi: 10.1186/1471-2156-12-21 (PMC3044674; doi:10.1186/1471-2156-12-21)

mtDNA

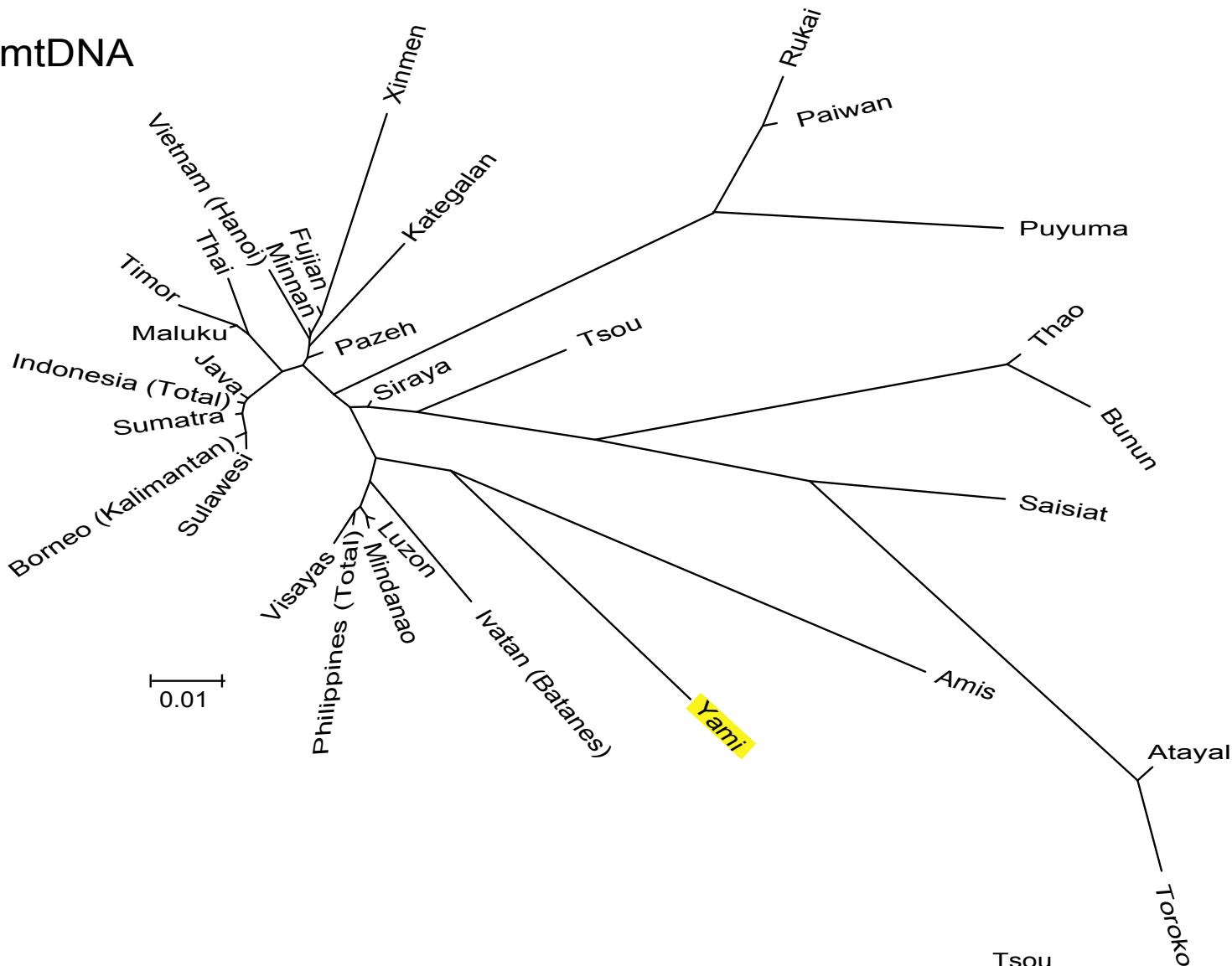

Y-STRs

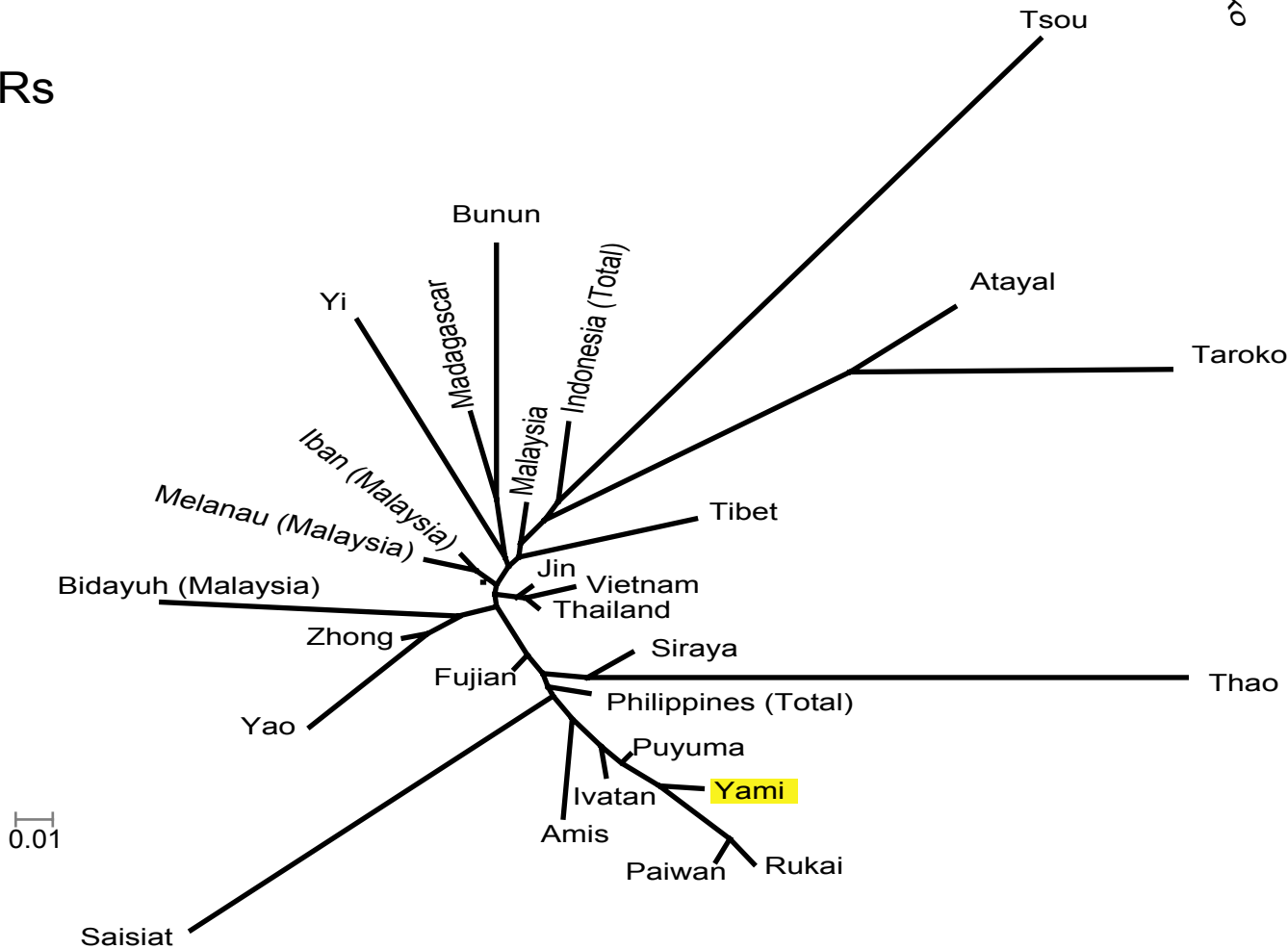

Supplement: Additional file 2 — Phylogenetic tree of populations of Taiwan, ISEA and MSEA using mtDNA (top) haplogroup frequencies (Fst distances) and Y-STR haplotypes frequencies (bottom). All mtDNA data information was obtained from the present study and from (Trejaut et al.; material in preparation). Y-STR data on Taiwan and ISEA was obtained from the present study and information for Mainland Southeast Asia populations was obtained from [39-43]. [file 1471-2156-12-21-S2.PDF]

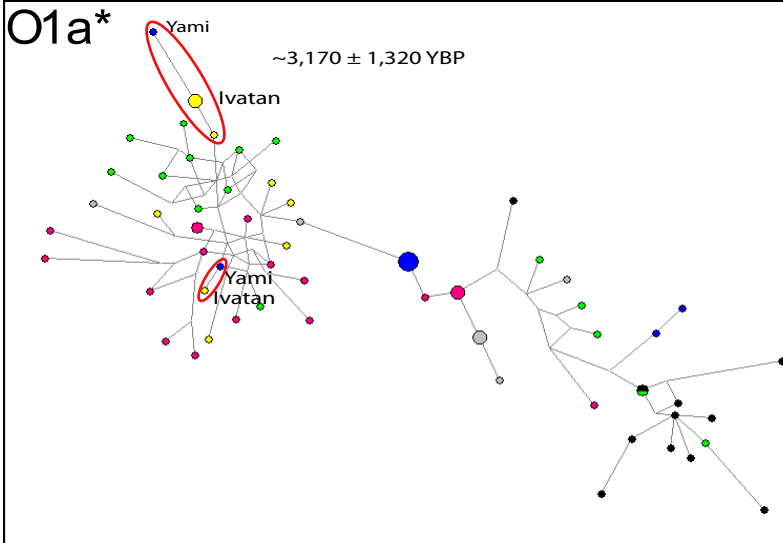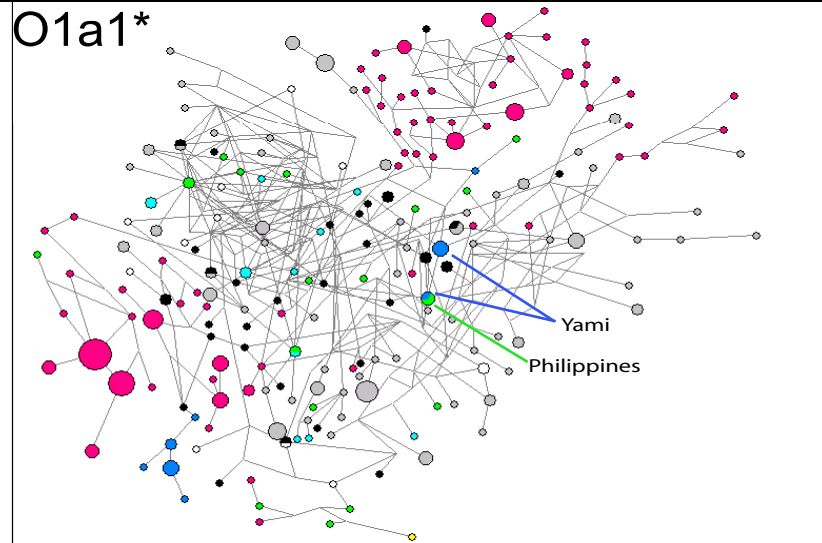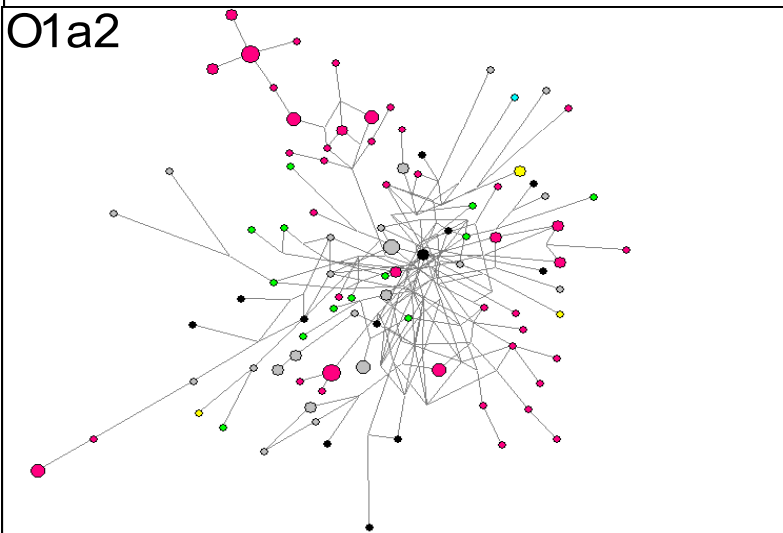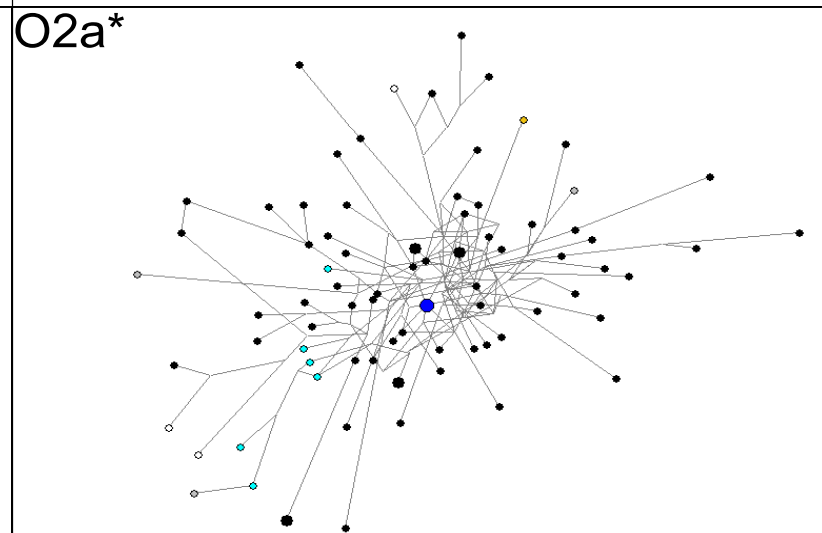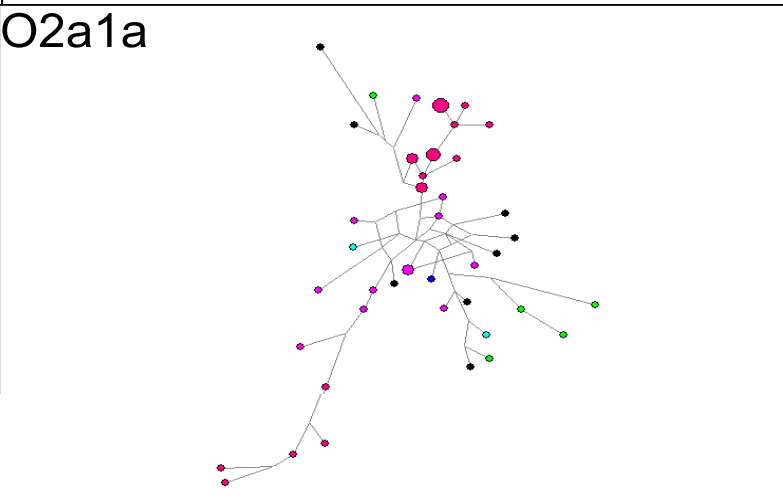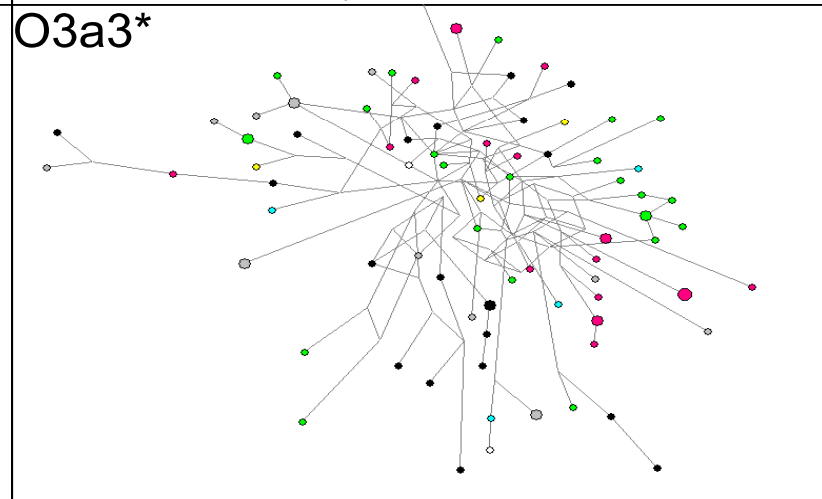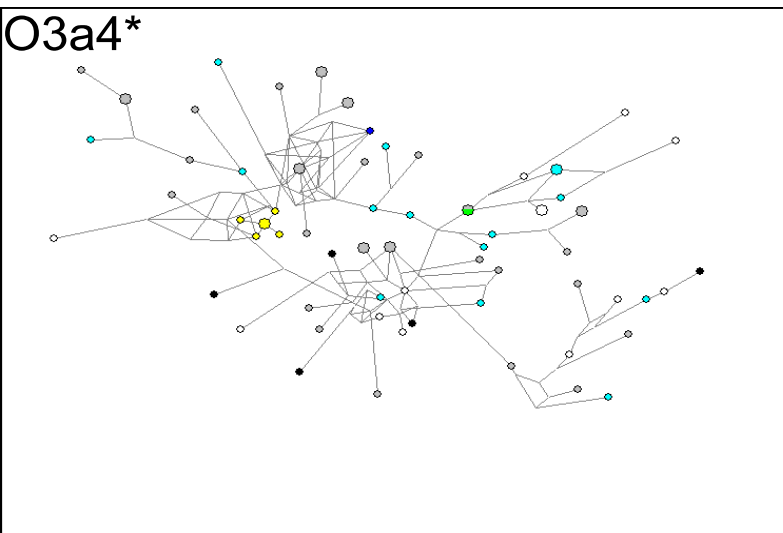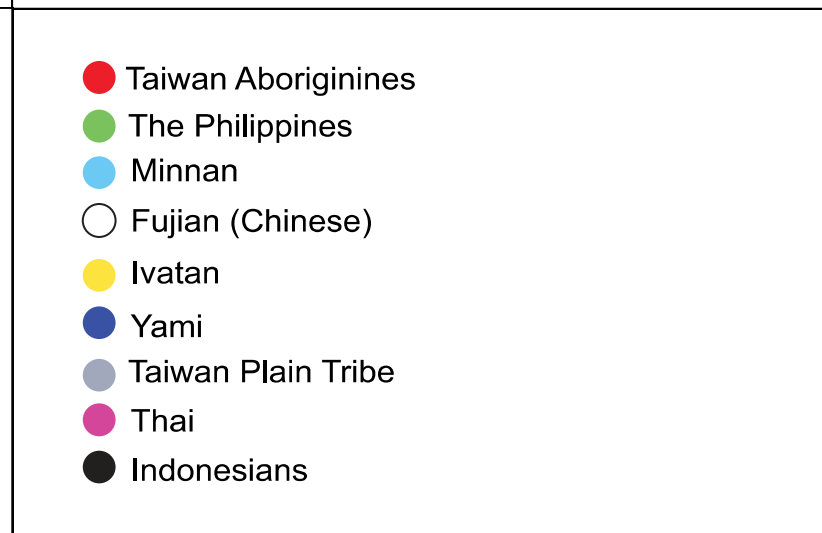

Supplement: Additional file 3 — Y-STR networks of Yami, Ivatan and other populations of ISEA and MSEA. Median-joining network for Taiwan, Southeast Asia and Island Southeast Asia of 16 Y-STR' variations within Haplogroup of O1, O2 and O3 (DYS19, DYS385a/b, DYS389I, DYS390, DYS390II, DYS391, DYS392, DYS393, DYS437, DY438, DYS439, DYS448, DYS456, DYS458, DYS635(YGATAC4), DYS635(YGATAH4). Circle areas are proportional to haplotype frequency and lines are the mutational differences between haplotypes. [file 1471-2156-12-21-S3.PDF]

## Wei and Liu Genealogy

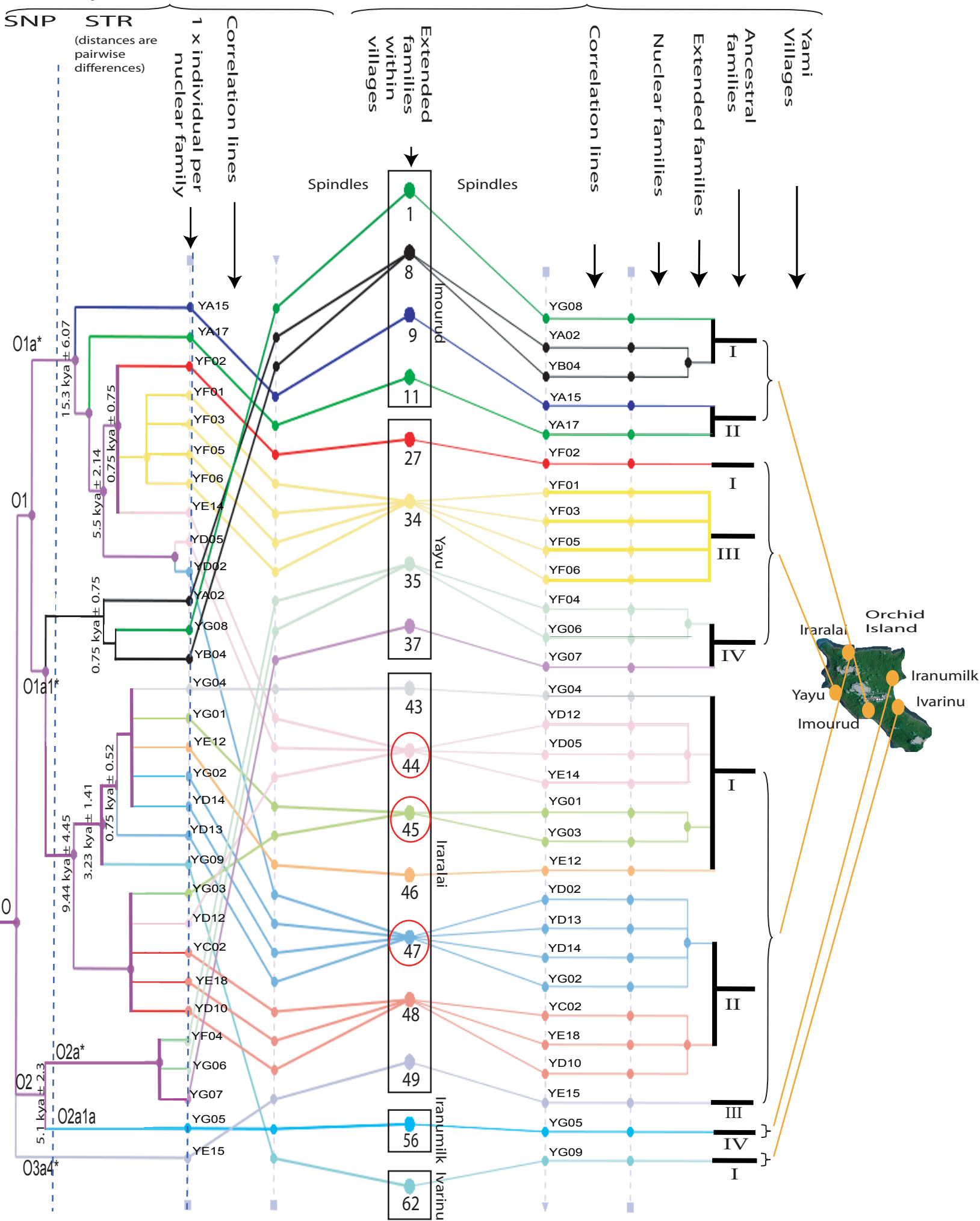

Supplement: Additional file 4 — Concordance between Yami NRY phylogenetic diversity (Y-SNP and Y-STR) and Wei and Liu ethnographic study of kinship (1962). Villages are represented by boxes and center brackets between Roman numerals. Each family is represented by a single Y-STR lineage along the correlation lines. The Correlation were obtain with the GenGIS program [68]. Concordance between Yami NRY phylogenetic diversity (Y-SNP and Y-STR) and the genealogy survey of Wei and Liu (1962) [22]. [file 1471-2156-12-21-S4.PDF]
