# Supplementary material for: Genetic affinities between the Yami tribe people of Orchid Island and the Philippine Islanders of the Batanes archipelago
Source: BMC Genet. 2011 Jan 31;12:21. doi: 10.1186/1471-2156-12-21 (PMC3044674; doi:10.1186/1471-2156-12-21)
Supplement: Additional file 5 — Possible settlement scenarios of Orchid Island and the Batanes archipelago from Taiwan or the Philippines. Scenario 1 is inspired from Ross linguistic study [2] and supports the "Out of Taiwan" model. The immediate ancestors of Proto Malayo-Polynesian speakers migrated out of Taiwan (~6,000 YBP to 4,000 YBP) to Orchid Island, the Batanes islands and Luzon, and developed languages specific to each regions (Figure 1). Scenario 2 is also inspired from Ross linguistic study [2]. In brief, the Proto Malayo-Polynesian origin is not located, but Northern Luzon is assumed to be a center of dispersion. As such, Orchid and Batanes islands could have been bypassed/ignored by the first migrants going from Taiwan to Northern Luzon (6,000 YBP to 4,000 YBP). Proto-Batanic languages would have developed during and after migrations from Luzon to the Batanes and Orchid islands (~3,000 YBP) where local languages later became more specific to Ivatan or Yami. Scenario 3 is based on genetics studies with first, a Bellwood-like expansion of people out of Taiwan ~4,000 years ago [10]. Secondly, Orchid and Batanes Islands could have been re-colonized from the south (as early as ~3,000 years ago, given the genetic estimates). Thirdly, later gene flow from Taiwan or Luzon would have affected the genetic profiles of people from Orchid or Batanes islands to look more like Taiwanese Aborigines or Filipinos respectively. Alternatively, the second stage could have been restricted to Ivatan who later extended their influence to Yami. This scheme is compatible with anthropological studies reporting that little to no external influence between Yami and Taiwan occurred from 1,500 YBP to 300 YBP [4]. The historically reported movement of people back and forth between Ivatan and Luzon during the 18th century typhoon and famine [23] most likely intensified Ivatan genetic affinity with Luzon and supports the last stage of this scenario. [file 1471-2156-12-21-S5.PDF]

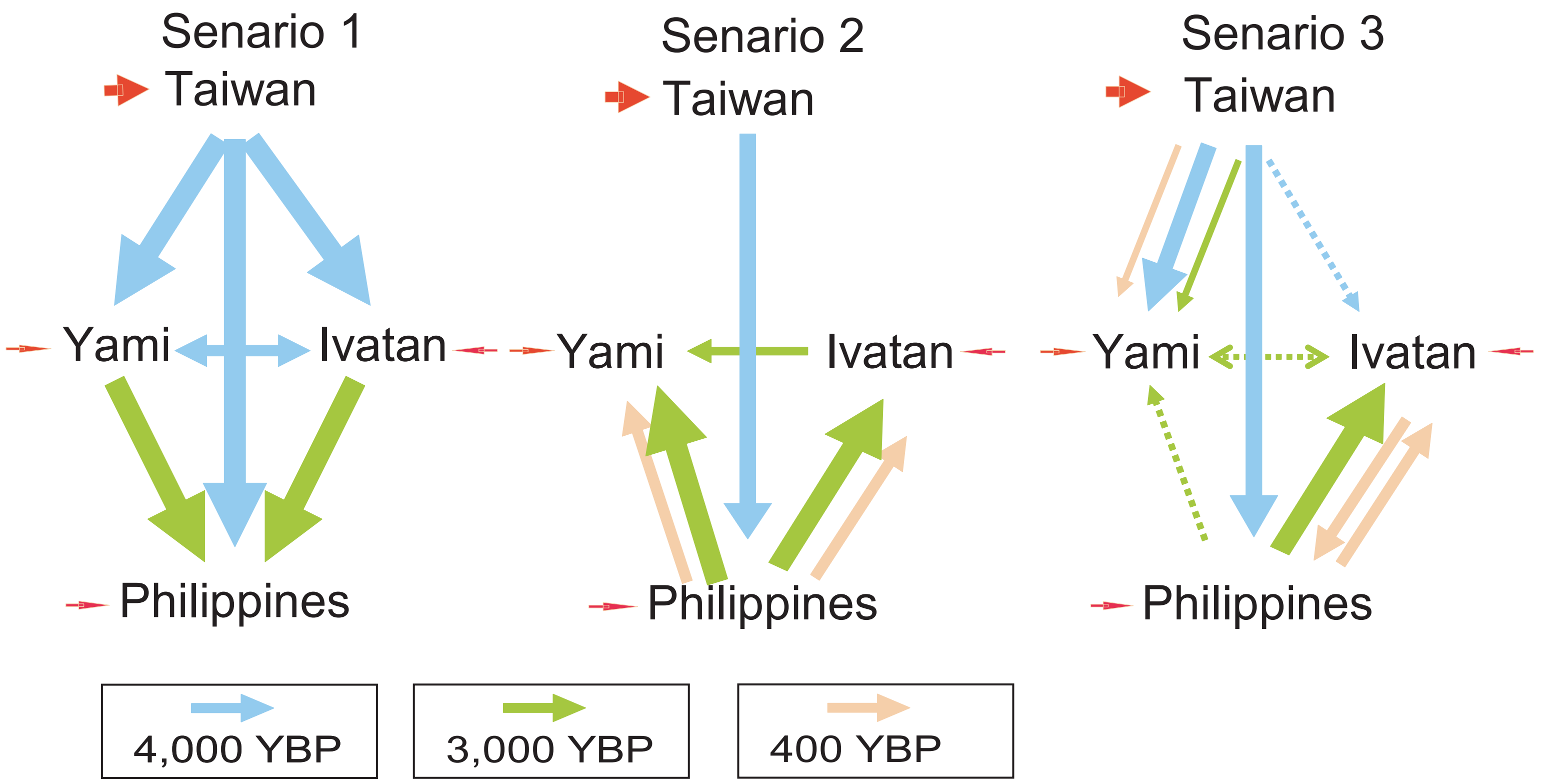

Thickness to dotted arrows indicates most to less important movements of migration

or or Non Han influence (Austronesian speakers)

Recent Han influence (400 YBP to present time)
